# Evaluation of Intraocular Lens Power Calculation Formulas for Highly Myopic Eyes: A Systematic Review and Network Meta-Analysis

**DOI:** 10.3390/jcm15155792

**Published:** 2026-07-24

**Authors:** Magí Vilaltella, Tonet Serés-Noriega, Pau Cid-Bertomeu, Valentín Huerva

**Affiliations:** 1 Department of Medicine and Surgery, Faculty of Medicine, University of Lleida, Carrer de Montserrat Roig, 2, 25008 Lleida, Spain; 2 Department of Ophthalmology, University Hospital Arnau de Vilanova, 25198 Lleida, Spain; 3 Department of Endocrinology and Nutrition, Centro Médico Milenium, 50006 Zaragoza, Spain; 4 Department of Ophthalmology, Hospital Sant Joan de Déu, 08243 Manresa, Spain

**Keywords:** myopia, cataract extraction, lenses, intraocular, biometry, refractive errors

## Abstract

**Background**: The most accurate intraocular lens power calculation formula for highly myopic eyes remains uncertain. The objective of this study was to perform a systematic review and network meta-analysis to compare and rank IOL power calculation formulas in eyes with an axial length greater than 26 mm. **Methods**: A literature search was conducted in PubMed, the Cochrane Library, Scopus, and Web of Science. The main outcome for comparison was the percentage of eyes with a refractive prediction error <0.25 diopters, <0.50 diopters, and <1.00 diopter. A network meta-analysis was performed, and Surface Under the Cumulative Ranking Curve values were calculated to rank formulas across the three prediction error thresholds. Forest plots were generated comparing all formulas with Barrett Universal II as the reference. **Results**: Ten studies were finally included. Based on Surface Under the Cumulative Ranking Curve values, Kane and Holladay 1 (with the original Wang–Koch axial length adjustment) ranked among the top three formulas across all prediction error thresholds. Hill-RBF 3.0, EVO, and Barrett Universal II ranked among the top three for the <0.25 diopters, <0.50 diopters, and <1.00 diopter thresholds, respectively. The Kane and Holladay 1 (with the original Wang–Koch axial length adjustment) formulas were the only ones showing odds ratios favouring their accuracy over Barrett Universal II across all prediction error thresholds in the forest plots, although differences were not statistically significant. **Conclusions**: The Kane and Holladay 1 (with the original Wang–Koch axial length adjustment) formulas showed the most consistent performance in highly myopic eyes. Hill-RBF 3.0, EVO, and Barrett Universal II also demonstrated high precision and may be considered reliable alternatives.

## 1. Introduction

Myopia is a common cause of visual impairment, with uncorrected myopia representing the leading cause of distance vision loss globally [1]. High myopia—defined as an axial length (AL) greater than 26 mm or a refractive error exceeding −6.00 diopters (D)—affects approximately 20% of individuals with myopia [2]. This condition is a known risk factor for cataract and other ocular diseases [2]. Consequently, early cataract surgery is frequently required in this population. Moreover, as surgical outcomes have improved, refractive lens exchange (RLE) has become more common, especially in highly myopic patients [3]. In this context, and with advances in equipment, surgical techniques and the increasing number of intraocular lens (IOL) power calculation formulas, achieving accurate refractive outcomes in cataract surgery for highly myopic eyes is now essential [4,5].

While most intraocular lens power calculation formulas demonstrate high accuracy in eyes with standard axial lengths, their performance often declines in eyes with axial lengths exceeding 26 mm [4,6,7,8]. Hyperopic prediction errors are commonly observed with increasing AL when using traditional IOL power calculation formulas, partly due to the challenge of accurately estimating the effective lens position (ELP) [4,9]. For this reason, many surgeons continue to target a mildly negative refractive outcome in eyes with axial myopia [8]. To compensate for the hyperopic shift, Wang et al. developed axial length adjustment methods for the Haigis, Hoffer Q, Holladay 1, and Holladay 2 formulas, known as the Wang–Koch formulas [10,11,12]. Moreover, several “new-generation” formulas have been introduced in recent years that substantially improve refractive outcomes in long eyes (e.g., Barrett Universal II, EVO, Kane, Hill-RBF) [13]. These formulas are primarily based on enhanced applications of Gaussian optics, artificial intelligence (AI), or a combination of both approaches [14].

Darcy et al. demonstrated that the Kane formula achieved the most accurate refractive outcomes overall and in the long axial length subgroup in a study involving 10,930 eyes, compared to other modern formulas [15]. In another study involving 370 highly myopic eyes, Cheng et al. reported that the Kane formula, along with Holladay 1_original W-K_ and Holladay 1_modified W-K_, achieved the best results compared to other new-generation formulas [16]. Conversely, Liu et al. found that the BU II formula yielded better refractive outcomes than the Holladay 1 and SRK/T formulas—both with the original and modified AL adjustments—in a cohort of 136 eyes with an axial length greater than 26 mm [17].

Despite the large number of studies on IOL power calculation formulas in eyes with long axial length, determining the most suitable formula for these patients remains challenging. The aim of this study is to perform a network meta-analysis to compare and rank IOL power calculation formulas in eyes with an axial length greater than 26 mm. The findings are intended to provide clinical guidance for surgeons in selecting the most appropriate IOL formula in highly myopic eyes.

## 2. Methods

This systematic review and meta-analysis was conducted in accordance with the Preferred Reporting Items for Systematic Reviews and Meta-Analyses (PRISMA) guidelines [18] and was not prospectively registered in any public registry. The completed PRISMA checklist is provided in the Supplementary Materials.

### 2.1. Search Strategy

Literature search was conducted in the following databases: PubMed, Cochrane Library, Scopus, and Web of Science. The Pubmed search strategy used the following terms: (“Cataract”[MESH] OR “Lenses, Intraocular”[MESH] OR “Phacoemulsification”[MESH] OR IOL[tw] OR “intraocular lens”[tw]) AND (calculat*[tw] OR formula*[tw]) AND (“Myopia”[MESH] OR “long eye”[tw] OR “high myopia”[tw] OR “long AL”[tw] OR “long axial length”[tw] OR “Axial Length, Eye”[MESH]). Only studies published in English or Spanish were considered, and the search included studies published from 2010 to August 2025.

Search strategy in Cochrane Library: cataract OR “intraocular lens” OR IOL OR phacoemulsification) AND (calculation OR formula) AND (myopia OR “high myopia” OR “long eye” OR “long axial length” OR “axial length”) (range 2010–2025).

Search strategy in Scopus: TITLE-ABS-KEY (cataract* OR “intraocular lens*” OR IOL OR phacoemulsif*) AND TITLE-ABS-KEY (calculat* OR formula*) AND TITLE-ABS-KEY (myopi* OR “high myopia” OR “long eye” OR “long AL” OR “long axial length”) (range 2010–2025. Subject area limited to: Medicine).

Search strategy in Web of Science: TS = (cataract* OR “intraocular lens*” OR IOL OR phacoemulsification) AND TS = (calculat* OR formula*) AND TS = (myopi* OR “high myopia” OR “long eye” OR “long AL” OR “long axial length”) (range 2010–2025).

### 2.2. Inclusion and Exclusion Criteria

Studies were included if they met the following criteria: (1) patients with an axial length greater than 26.00 mm undergoing uneventful, sutureless phacoemulsification cataract surgery with in-the-bag insertion of a monofocal intraocular lens; (2) biometry performed using an optical biometer; (3) evaluation of at least two of the following target formulas: Barrett Universal II [19], Kane [20], EVO [21], Hill RBF 2.0, Hill RBF 3.0 [22], Pearl-DGS [23], Olsen [24], Haigis [25], Holladay 2, Holladay 1 [26], SRK/T [27], SRK/T with the original or modified Wang–Koch AL adjustment (SRK/T_original W-K_, SRK/T_modified W-K_), or Holladay 1 combined with the original or modified Wang–Koch AL adjustment (Holladay 1_original W-K_, Holladay 1_modified W-K_) [10,11,12]; and (4) studies published within the last 15 years. Exclusion criteria were as follows: (1) implantation of multifocal or toric IOLs; (2) history of previous refractive or intraocular surgery; (3) IOL constant optimization for the study group or adjustment of mean error (ME) to zero not applied; (4) percentage of eyes with a predictive error (PE) within ±0.25 D, ±0.50 D and ±1.00 D not provided; (5) studies conducted in pediatric populations; and (6) review articles or letters to the editor.

### 2.3. Data Extraction and Quality Assessment

Duplicated studies were removed first. Two researchers (MV and PC) independently screened the titles and abstracts of the remaining records to exclude those that did not meet the eligibility criteria. Any discrepancies were resolved by a third author (VH). A full-text review of the potentially eligible studies was then performed by one researcher (MV) for final selection. The following data were extracted from each included study: year of publication, first author, number of eyes, types of IOLs, patient characteristics (mean age, mean AL, mean Keratometry (K), mean Anterior Chamber Depth (ACD), mean Lens Thickness (LT)), IOL power calculation formulas evaluated, and main outcomes analyzed (the percentage of eyes with a prediction error within ±0.25 D, ±0.50 D, and ±1.00 D).

The quality of evidence of the included studies was assessed using a modified checklist derived from the QUADAS-2 (Quality Assessment of Diagnostic Accuracy Studies) tool, which has previously been applied in other systematic reviews and meta-analysis comparing IOL power calculation formulas [28,29,30]. This tool evaluates the risk of bias and concerns regarding applicability for each study by analyzing four domains: patient selection, index test, reference standard, and flow and timing [31]. The modifications in the original QUADAS 2 checklist [32] were made to account for the specifical methodological characteristics of studies evaluating refractive prediction accuracy rather than diagnostic test performance.

### 2.4. Statistical Analysis

The main outcomes analyzed were the percentages of eyes within a refractive error of ±0.25 D, ±0.50 D, and ±1.00 D. First, a traditional pairwise meta-analysis was conducted by calculating the odds ratio (OR) for all possible direct comparisons between formulas. For each comparison, statistical heterogeneity was assessed using the I^2^ statistic; when heterogeneity exceeded 50%, a random-effects model was used to estimate the pooled odds ratio, whereas a fixed-effects model was applied when heterogeneity was 50% or lower [29].

Second, a network meta-analysis was performed, allowing all formulas to be compared with each other by integrating both direct and indirect evidence. This approach enables the comparison of two formulas even in the absence of head-to-head comparisons in individual studies. Inconsistency between direct and indirect evidence was assessed using node-splitting analyses for each comparison. If no inconsistency was detected, a consistency model was applied to estimate the relative effects of all formulas, with fixed- or random-effects models applied according to the degree of heterogeneity (I^2^) [28,29,30]. Moreover, ranking probabilities and the surface under the cumulative ranking curve (SUCRA) were calculated to estimate the probability of each formula being ranked at each possible position and to summarize the overall ranking of the formulas [29,30]. SUCRA is expressed as a percentage; higher SUCRA values indicate better positions in the overall ranking.

Formulas evaluated in only one study were excluded from the pairwise and network meta-analysis to ensure sufficient data for valid comparisons and to avoid disconnected nodes in the network structure.

Comparison-adjusted funnel plots were employed to evaluate the risk of publication bias [30,33]. Visual symmetry in the plots for any of the outcomes assessed was interpreted as indicative of a relatively low risk [29]. Statistical analyses were performed using Stata (Statistical Software: Release 17. College Station, TX, USA: StataCorp LLC).

## 3. Results

### 3.1. Literature Selection

**Figure 1:**
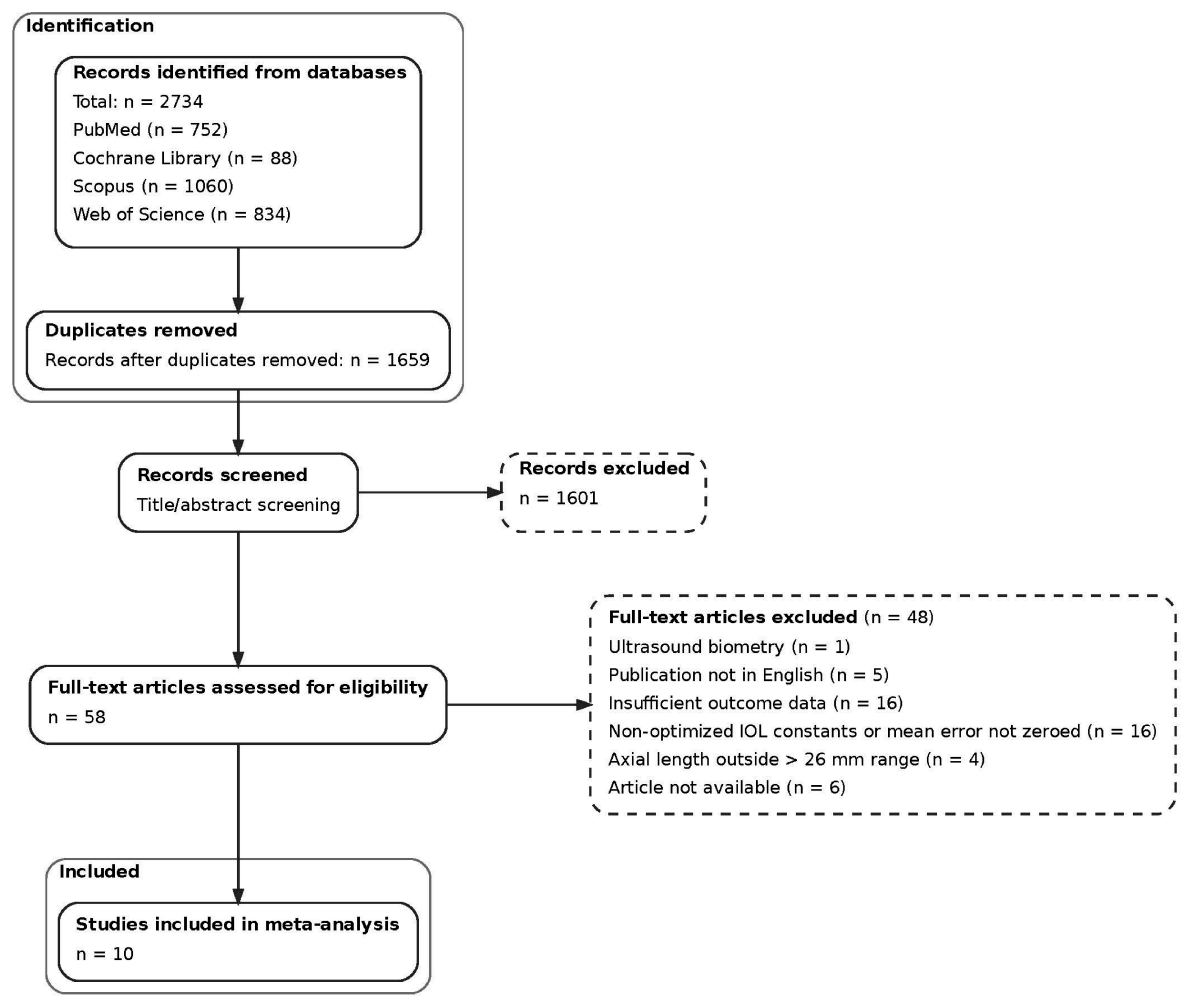
Study Selection Flow Diagram.

The initial search across selected databases yielded a total of 2734 records: 752 from PubMed, 88 from the Cochrane Library, 1060 from Scopus, and 834 from Web of Science. After removing duplicates, 1659 unique records were retained. Title and abstract screening led to the exclusion of 1601 records. Subsequently, 58 full-text articles were assessed for eligibility. Of these, 48 were excluded for the following reasons: use of ultrasound biometry (n = 1), publication not in English (n = 5), insufficient outcome data (n = 16), use of non-optimized IOL constants or failure to zero out the mean error prior to analysis (n = 16), axial length outside the >26 mm range (n = 4), and article not available (n = 6). Ten studies met the eligibility criteria and were finally included in the meta-analysis. The flow diagram of the study selection process is presented in Figure 1. The characteristics of the included studies are summarized in Table 1.

### 3.2. Network Plot

**Figure 2:**
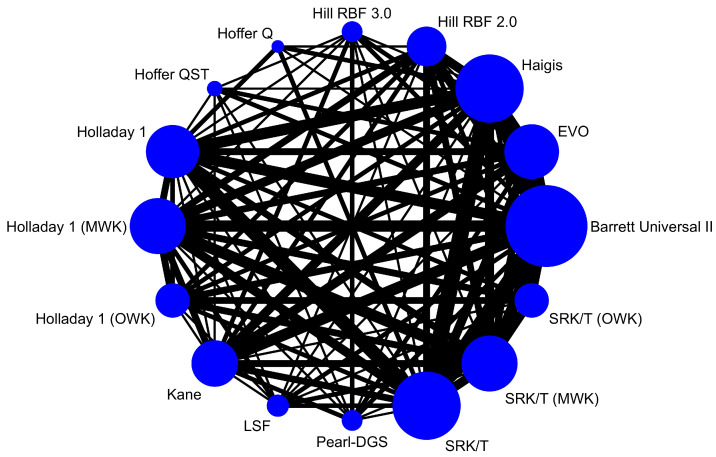
Network plot of all comparisons. SRK/T (OWK): SRK/T_original W-K_; SRK/T (MWK): SRK/T_modified W-K_; Holladay 1 (MWK): Holladay 1_modified W-K_; Holladay 1 (OWK): Holladay 1_original WK_.

Figure 2 presents the network plot of direct comparisons among all formulas. Each node represents a formula, with its size proportional to the number of eyes included in the analysis for that formula. Edges between nodes indicate direct comparisons, and the width of each line reflects the number of studies contributing to that comparison.

### 3.3. Direct Pairwise Meta-Analysis

Additional Files S1–S3 present the results of direct pairwise comparisons between the formulas. For the proportion of eyes with a prediction error <0.25 diopters, the odds ratios indicated superior performance of the Hill-RBF 3.0, Kane, and Holladay 1_original W-K_ formulas. However, statistically significant differences were observed only in the following comparisons: Holladay 1_original W-K_ vs. SRK/T_modified W-K_, Kane vs. LSF, and Kane vs. SRK/T_modified W-K_. For prediction errors <0.50 D, Holladay 1_original W-K_ and Kane yielded a higher percentage of eyes within this range, although statistical significance was observed only for Kane vs. LSF. In the <1.00 D category, Holladay 1_original W-K_, Kane, and Barrett Universal II also showed higher percentages than the other formulas, but none of the differences were statistically significant. The direct meta-analysis revealed minimal heterogeneity (I^2^ < 50%) across most comparisons, except for a few involving the LSF formula. Consequently, a fixed-effects model was applied in the majority of cases to estimate the pooled odds ratios.

### 3.4. Pairwise Network Meta-Analysis (NMA)

Detailed results of the pairwise network meta-analysis are presented in Additional Files S4–S6. The estimated pooled odds ratios derived from direct and indirect comparisons indicate superiority of the BU II, EVO, Kane, and Holladay 1_original W-K_ formulas in the percentage of eyes within the different prediction error thresholds (<0.25 D, <0.50 D, and <1.00 D), with statistically significant differences compared with several other formulas. Hill RBF 3.0 and Holladay 1_modified W-K_ also yielded multiple significant differences in the <0.25 D and <0.50 D prediction error groups, whereas Hill RBF 2.0 also stood out in the <0.50 D and <1.00 D groups, with several statistically significant comparisons.

### 3.5. Forest Plots (NMA)

**Figure 3:**
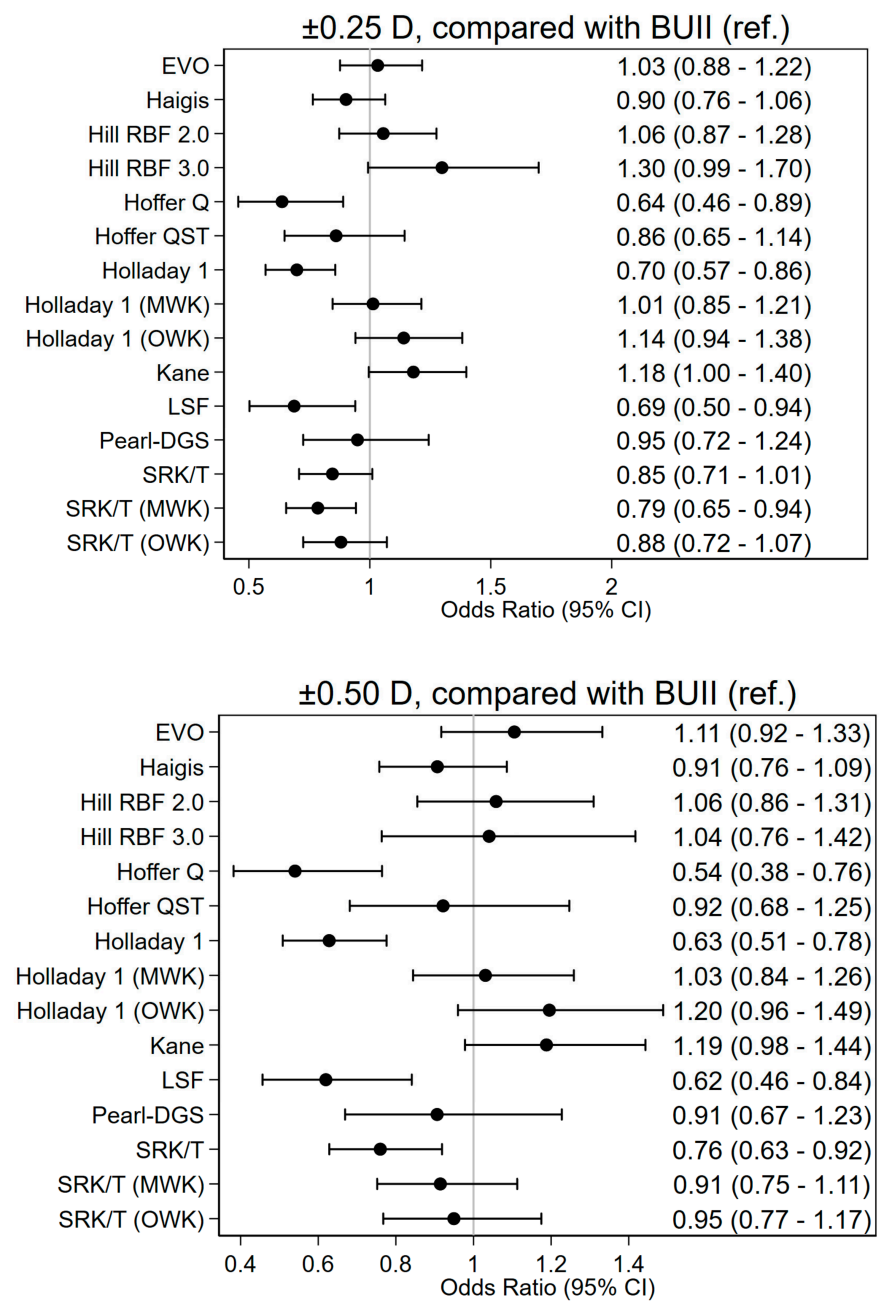

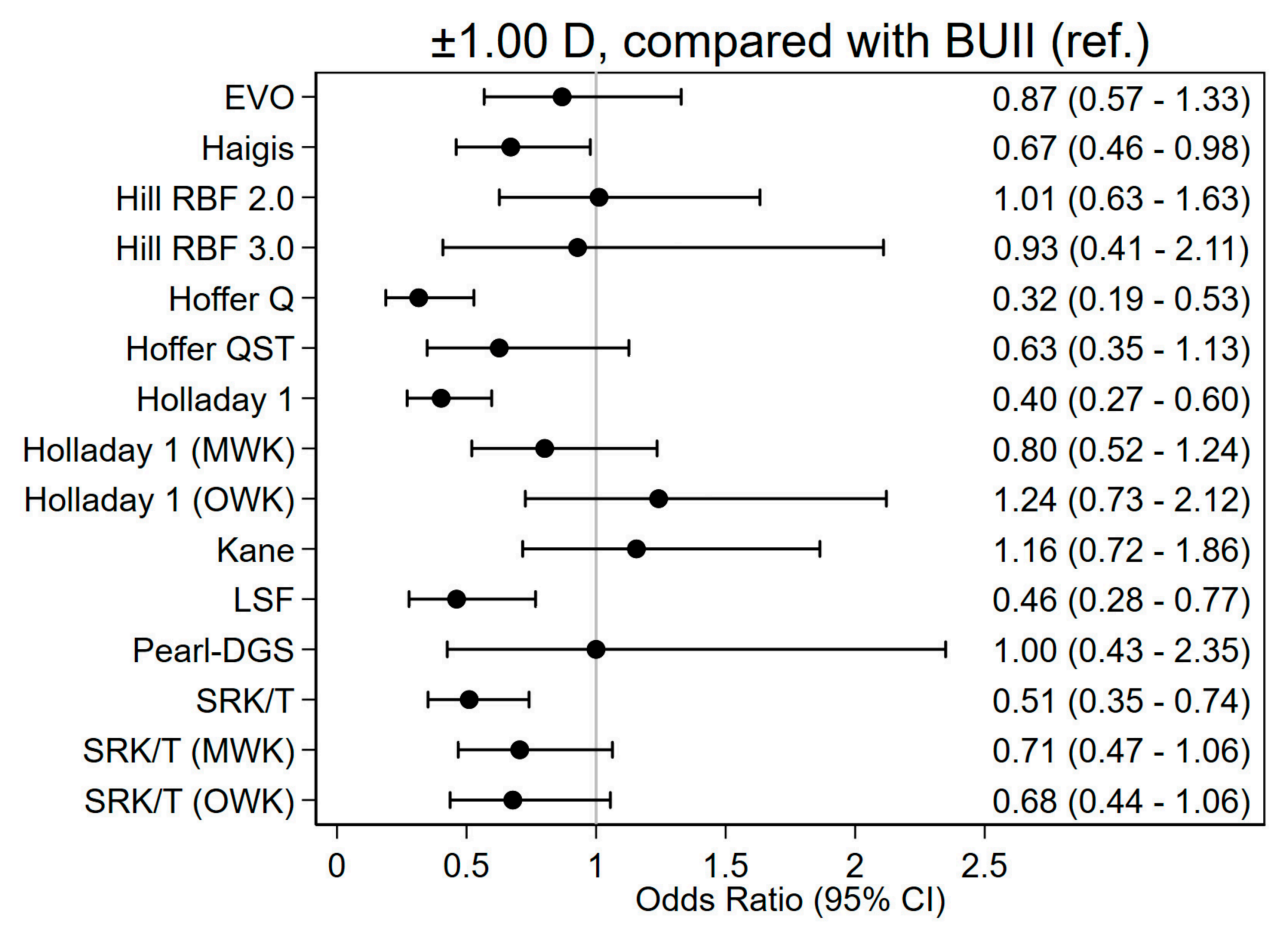
Forest plots of the Network Meta-Analysis comparing all formulas with Barrett Universal II as the reference for the percentage of eyes with a refractive prediction error ≤0.25 D, ≤0.50 D, and ≤1.00 D. CI: confidence interval; D: diopter; ref.: reference; SRK/T (OWK): SRK/T_original W-K_; SRK/T (MWK): SRK/T_modified W-K_; Holladay 1 (MWK): Holladay 1_modified W-K_; Holladay 1 (OWK): Holladay 1_original W-K_.

Forest plots presented in Figure 3, comparing all formulas to Barrett Universal II, showed that EVO, Hill-RBF 2.0, Hill-RBF 3.0, Holladay 1_modified W-K_, Holladay 1_original W-K_, and Kane achieved a higher percentage of eyes within the <0.25 D and <0.50 D prediction error thresholds. In the <1.00 D prediction error category, only Hill-RBF 2.0, Holladay 1_original W-K_, and Kane demonstrated superiority over Barrett. However, none of these differences reached statistical significance.

### 3.6. Node-Split and Heterogeneity (NMA)

Results of the node-splitting analysis for inconsistency are summarized in Additional Files S7–S9. No evidence of inconsistency (*p* > 0.05) between direct and indirect estimates was found in the vast majority of comparisons. Moreover, the I^2^ statistic indicated minimal heterogeneity across most comparisons. Consequently, a fixed-effects consistency model was applied to estimate the relative effects of all formulas in the network meta-analysis.

### 3.7. SUCRA and Ranking Probabilities (NMA)

**Figure 4:**
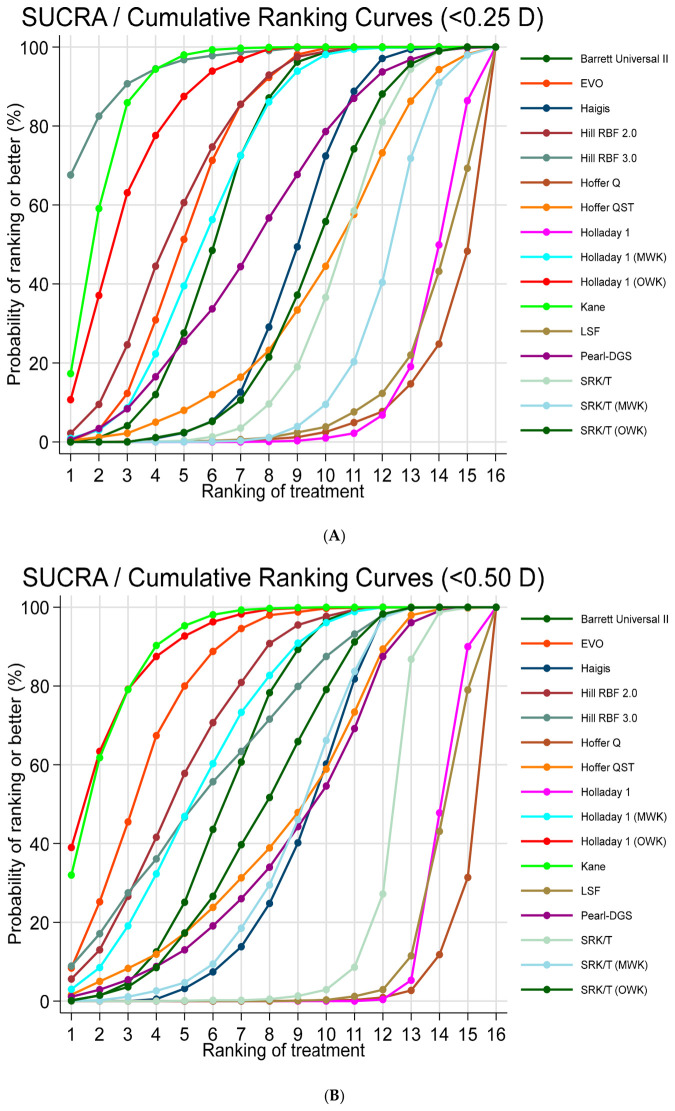

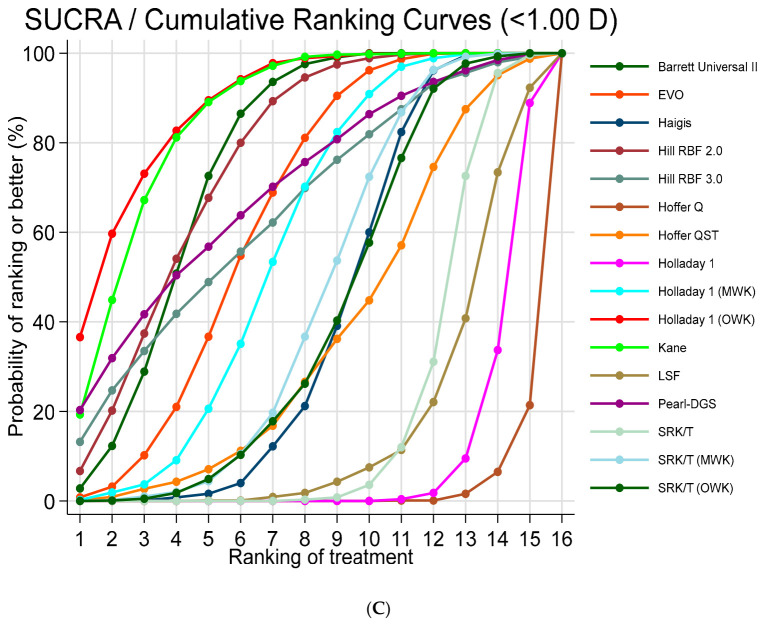
(**A**). Cumulative ranking curves of all formulas for the percentage of eyes with a refractive prediction error ≤0.25 DP. (**B**). Cumulative ranking curves of all formulas for the percentage of eyes with a refractive prediction error ≤0.50 DP. (**C**). Cumulative ranking curves of all formulas for the percentage of eyes with a refractive prediction error ≤1.00 DP.

Figure 4 displays the cumulative ranking curves for all formulas across the three prediction error thresholds (<0.25 D, <0.50 D, and <1.00 D). The area under each curve corresponds to the respective SUCRA value. Exact SUCRA values for each formula are provided in Additional Files S10–S12.

For the <0.25 D group, Hill-RBF 3.0 had the highest probability of being the best-performing formula, with a SUCRA value of 95.3%, followed by Kane (89.9%) and Holladay 1_original W-K_ (85.0%). Regarding ranking probabilities, Hill-RBF 3.0 was most likely to rank first (67.4%), followed by Kane (16.7%) and Holladay 1_original W-K_ (11.7%).

In the <0.50 D group, Kane achieved the highest SUCRA value (90.5%), closely followed by Holladay 1_original W-K_ (90.3%) and EVO (80.1%). The probabilities of ranking first were 38.7% for Holladay 1_original W-K_, 33.1% for Kane, and 10.5% for Hill-RBF 3.0.

For the <1.00 D threshold, the formulas with the highest SUCRA values were Holladay 1_original W-K_ (88.7%), Kane (85.8%), and Barrett Universal II (76.5%). However, the highest probabilities of ranking first were observed for Holladay 1_original W-K_ (35.1%), Kane (19.9%), and Pearl-DGS (19.8%).

### 3.8. Risk of Publication Bias and Quality Assessment

Comparison-adjusted funnel plots are shown in Additional File S13. Visual inspection of the plots for the three prediction error thresholds (<0.25, <0.50, and <1.00 diopters DP) revealed no relevant asymmetry, suggesting a low risk of publication bias or small-study effects.

Detailed results of the application of the modified QUADAS-2 tool to the included studies are presented in Additional File S14. Regarding the ‘Patient Selection’ domain, two studies did not specify the method of patient enrolment. With respect to the ‘Reference Standard’ domain, one study did not report the type of postoperative refraction performed. Finally, in the ‘Flow and Timing’ domain, three studies did not specify whether postoperative refraction was performed consistently across all patients. In terms of applicability concerns, all studies were judged to be of high quality.

## 4. Discussion

Although intraocular lens power calculation yields satisfactory results in most eyes with standard axial lengths, it remains challenging in highly myopic eyes [5]. Therefore, we conducted a meta-analysis to systematically compare the most commonly used formulas in eyes with an axial length of 26.00 mm or greater.

The Kane and Holladay 1_original W-K_ formulas appear to be superior to the others, as they both ranked among the top three SUCRA values for the percentage of eyes with a prediction error <0.25 D, <0.50 D, and <1.00 D. Moreover, these were the only formulas that showed favourable odds ratios compared to Barrett Universal II across all three PE groups in the forest plots, although the differences did not reach statistical significance. Hill-RBF 3.0, EVO, and Barrett Universal II should also be considered among the most accurate formulas, as they likewise ranked within the top three—based on SUCRA values—in the <0.25 D, <0.50 D, and <1.00 D thresholds, respectively.

The Kane formula ranked second in the percentage of eyes with a PE <0.25 D and <1.00 D and first in the <0.50 D threshold. Developed by Jack X. Kane, it integrates theoretical optics, regression techniques, and artificial intelligence and was derived from a large dataset of 30,000 selected refractive cataract cases [14,15]. The required parameters for calculating the Kane formula include AL, ACD, K, and sex; LT and central corneal thickness (CCT) are optional. In a study involving 10,930 eyes from the UK National Health Service, the Kane formula demonstrated superior accuracy overall and across all axial length subgroups (<22 mm, 22–26 mm, and >26 mm) when compared with other formulas, including Hill-RBF 2.0, Olsen, and Barrett Universal II [15]. In another study conducted by Lin et al., which included 175 eyes with long axial length (>26.00 mm), the Kane formula yielded the lowest median absolute error and the highest percentage of eyes with a prediction error <0.25 D, compared to EVO, BU II, Haigis, and SRK/T [5].

The Holladay 1_original W-K_ formula ranked third, second and first in the percentage of eyes with a prediction error <0.25 D, <0.50 D and <1.00 D, respectively. The Wang-Koch AL adjustment method was originally proposed by Wang et al. as a method to reduce hyperopic outcomes in long eyes for the Haigis, Hoffer Q, Holladay 1 and SRK/T formulas [10]. The method was later modified for the Holladay 1 and SRK/T formulas by incorporating ULIB lens constants (instead of the manufacturer’s) and converting manifest refraction to 6 m, in order to reduce myopic errors [11]. Cheng et al. reported that the Holladay 1 formula—using both the original and modified AL adjustments—along with the Kane formula, achieved the best refractive outcomes in terms of mean absolute error (MAE) and median absolute error (MedAE) in a cohort of 370 highly myopic eyes, compared to other formulas including Barrett Universal II, EVO, and Hill-RBF 2.0 [16].

The Hill-RBF 3.0 formula achieved the highest SUCRA score (95.3%) for the percentage of eyes with a prediction error <0.25 D, followed by the Kane formula (89.9%) and the Holladay 1_original W-K_ formula (85.0%). Hill-RBF is a purely data-driven method that combines artificial intelligence and regression analysis based on a large dataset of post-refractive outcomes—12,000 cases in version 2.0 and 30,000 in version 3.0 [14,38]. As its predictions rely primarily on empirical data, if the biometric characteristics of an eye do not match those in the Hill-RBF database, the prediction may be less accurate, and an ‘out-of-bounds’ notification is generated [38]. In a study conducted by Mo et al., involving 391 highly myopic eyes (AL > 26.00 mm), Hill-RBF 3.0 achieved the best refractive outcomes compared to 14 modern and 4 traditional IOL formulas [39]. Similarly, Stopyra et al. reported that, in a cohort of extremely long eyes (AL > 30 mm), Hill-RBF 3.0 yielded the lowest root mean square absolute error (RMSAE) compared to six other AI-based formulas, including Kane [40].

The EVO formula yielded the third-highest SUCRA value (80.1%) for the percentage of eyes with a prediction error <0.50 D, following the Kane (90.5%) and Holladay 1_original W-K_ (90.3%) formulas. The EVO formula is a thick-lens formula based on the ‘theory of emmetropization’ [14,41]. The original version (1.0) was introduced in June 2016, whereas version 2.0, released in June 2019, incorporated improvements in prediction accuracy and additional functionalities [42]. Notably, all studies included in the present network meta-analysis evaluated EVO version 2.0. The biometric parameters required for its calculation are K, AL and ACD, while LT and CCT are optional. Lin et al. concluded that the EVO and Kane formulas achieve better refractive outcomes in patients with high axial myopia compared to Barrett Universal II, Haigis, and SRK/T [5]. Moreover, Li et al. demonstrated that the EVO, Barrett, Kane, and Wang–Koch AL–adjusted formulas exhibit comparable accuracy for IOL power calculation in eyes with axial lengths greater than 30 mm [43].

Barrett Universal II achieved the third-highest SUCRA score (76.5%) for the percentage of eyes with a prediction error <1.00 D, following the Holladay 1_original W-K_ (88.7%) and Kane (85.8%) formulas. Barrett Universal II is widely considered one of the most accurate IOL formulas currently in use and is highly popular among cataract surgeons [29,38]. It is a vergence-based formula that employs a unique theoretical model to predict the effective lens position, which is quite distinct from the models used in other formulas [14]. Several studies have demonstrated that Barrett Universal II can achieve refractive outcomes comparable to newer-generation IOL formulas [7,8,44] and substantially better results than third- and fourth-generation formulas [45], in highly myopic eyes.

We performed a network meta-analysis in our study, which offers several advantages over traditional pairwise meta-analysis. It integrates both direct and indirect evidence, providing estimates for comparisons that may not have been evaluated in head-to-head studies and improving statistical power and precision by pooling information across the network. Moreover, while pairwise meta-analysis compares only two treatments at a time, network meta-analysis allows multiple interventions to be compared within a single analytical framework. It also enables ranking of interventions based on their probability of being the most effective, thereby offering a more comprehensive overview of the available evidence. In this sense, SUCRA was used as the main efficacy outcome. It represents the surface under the cumulative ranking curve and provides a summary measure of the overall ranking performance of each formula across all possible positions.

Potential effect modifiers should be similarly distributed across the different comparisons; otherwise, indirect comparisons may be biassed and the validity of the network meta-analysis may be compromised [46]. In our study, the transitivity assumption appeared to be satisfied, as suggested by low heterogeneity and the absence of significant inconsistency across most comparisons.

To our knowledge, only two other network meta-analyses have specifically evaluated the accuracy of IOL power calculation formulas in highly myopic eyes [29,47], which highlights the clinical relevance of the present study. One of these, the recent network meta-analysis by Zhou et al. evaluated 2430 eyes from 12 studies with axial lengths >26 mm and identified XGBoost, Hill-RBF, and Kane as the highest-ranked formulas for IOL power calculation in highly myopic eyes [47]. They also reported that these AI-based formulas were significantly more accurate than traditional vergence formulas. Consistent with these findings, Kane ranked among the best-performing formulas in our network meta-analysis, whereas XGBoost could not be evaluated because it was not assessed in any of the eligible studies. In addition, our pairwise network meta-analysis showed that, besides Hill-RBF and Kane, EVO, Barrett Universal II, and Holladay 1 (with the original and modified Wang-Koch AL adjustment) also demonstrated significantly better performance than traditional vergence formulas. Furthermore, Holladay 1_original W-K_ ranked among the top three formulas across all three prediction error thresholds. These differences between the two studies may be explained, at least in part, by methodological differences, particularly the inclusion of only studies using specifically optimized IOL constants, which resulted in a different set of eligible studies.

This study has several limitations. First, some of the included studies in the meta-analysis used data from both eyes of the same patient. In IOL power calculation studies, it is generally recommended to include only one eye per patient, as ocular biometric measurements tend to be highly correlated between fellow eyes. Including both eyes can lead to data clustering and may introduce bias [48,49]. In this regard, none of the studies that included bilateral eyes adjusted their primary statistical analyses for inter-eye correlation. However, when pooled, only 226 of the 1531 eyes included in this meta-analysis were from bilateral cases, representing 14.7% of the total sample. We therefore consider that the impact of this limitation on the overall estimates is likely to be limited. Second, in six of the ten included studies, biometry was performed using the IOL Master 500, which does not measure LT or CCT. Although these parameters are not mandatory for any of the formulas assessed in the meta-analysis, they are optional inputs for several modern formulas, and their absence may have limited the ability of these formulas to achieve optimal predictive accuracy [15]. Therefore, this limitation should be considered when interpreting the comparative ranking of the formulas included in the present network meta-analysis.

We did not compare mean absolute errors across formulas because relatively few studies reported measures of dispersion, precluding the inclusion of this outcome in the meta-analysis. Additionally, although several methods have been proposed to pool medians in meta-analyses [50], their application also requires detailed reporting of distributional measures (such as interquartile ranges or ranges), which were likewise insufficiently reported in the included studies. Therefore, median absolute errors could not be quantitatively compared. The reported MAE and MedAE values from the individual studies are provided in Additional File S15 for interested readers.

Finally, to preserve the internal validity of the network, formulas reported in only a single study were excluded. Including such formulas could have introduced bias by amplifying underpowered or non-generalizable findings. Moreover, formulas lacking replication across studies may not be suitable for robust comparative analysis. However, this methodological decision also limited the inclusion of some promising formulas, such as Cooke K6, which has demonstrated excellent performance in extremely long eyes [34].

## 5. Conclusions

In conclusion, our investigation suggests that the Kane and Holladay 1_original W-K_ formulas provide superior accuracy in predicting refractive outcomes in highly myopic eyes. Additionally, Hill-RBF 3.0, EVO, and Barrett Universal II also demonstrate high overall precision and should be considered reliable alternatives. Nevertheless, this remains a subject of ongoing debate, and further original clinical studies with sufficiently large sample sizes are necessary to confirm these findings.

## Figures and Tables

**Table 1:** Characteristics of the included studies.

Study	Year	Number Eyes	Types IOLs	Mean Age ± SD (y)	Mean AL ± SD	Mean K ± SD (D)	Mean ACD ± SD	Mean LT ± SD	IOL Formulas
Bernardes J et al. [4]	2021	82	Acrysof MA60MA	57.6 ± 8.7	30.89 ± 1.85	44.26 ± 1.76	3.46 ± 0.35	N/A	A, C, D, I, K, L, N, O
Wan K et al. [8]	2019	127	Tecnis ZCB00, AMO AR40E, Alcon SN60WF, Alcon SA60WF, Alcon SA60AT, Alcon MA60M, B&L enVista MX60	65.8 ± 9.1	27.72 ± 1.59	43.47 ± 1.41	3.41 ± 0.30	N/A	A, C, D, F, H, N
Vilaltella M et al. [7]	2025	72	Acrysof SN60WF, Acrysof MN60MA (+D), Acrysof MN60MA (−D), B&L SZ-1 EyeCee One	66 ± 10.76	27.37 ± 1.53	43.48 ± 1.48	3.46 ± 0.33	N/A	A, B, C, E, H, I, K, M, N, O
Cheng H et al. [16]	2020	370	Acrysof SN60WF, AR40e/E/M, ZA9003, Adapt AO, 920H	59.3 ± 12.6	28.98 ± 2.23	N/A	3.53 ± 0.41	N/A	A, B, C, D, I, J, K, O, P
Lin L et al. [5]	2021	175	N/A	61.78 ± 11.43	28.66 ± 2.028	44.13 ± 1.663	3.314 ± 0.445	4.353 ± 0.412	A, B, C, K, N
Mo E et al. [34]	2023	236	A1-UV	59 [53, 68] ^a^	31.4 [30.67, 32.39] ^a^	N/A	3.44 ± 0.35	4.48 ± 0.39	A, B, E, G, K, M
Liu J et al. [17]	2019	136	Akreos Adapt AO, AR40e/E/M, 920H, SA60AT/SN60WF, Softec I/Softec HD	61 ± 11	28.85 ± 2.02	44.48 ± 1.82	3.65 ± 0.40	N/A	A, C, D, H, I, J, N, O, P
Whang W et al. [35]	2025	61	Rayner Superflex 920H	56.61 ± 10.50	33.23 ± 1.58	44.48 ± 1.74	3.70 ± 0.38	N/A	A, B, C, G, H, N
Zhang J et al. [36]	2020	164	B&L enVista MX60	59.38 ± 13.25	28.78 ± 2.32	N/A	3.49 ± 0.37	4.38 ± 0.46	A, B, H, I, J, L, N, O, P
Zhang J et al. [37]	2019	108	Rayner 920H	57.46 ± 12.56	29.15 ± 2.31	43.85 ± 1.62	3.51 ± 0.39	N/A	C, F, H, N

A: Barrett Universal II; B: EVO 2.0; C: Haigis; D: Hill RBF 2.0; E: Hill RBF 3.0; F: Hoffer Q; G: Hoffer QST; H: Holladay 1; I: Holladay 1_modified W-K_; J: Holladay 1_original W-K_; K: Kane; L: LSF; M: Pearl-DGS; N: SRK/T; O: SRK/T_modified W-K_; P: SRK/T_original W-K_; SD: standard deviation; y: years; AL: axial length; K: keratometry; D: diopters; ACD: anterior chamber depth; LT: lens thickness. ^a^ Reported as median [IQR].

## Data Availability

All data generated or analyzed during this study are included in this published article and its Supplementary Information Files. The original data were extracted from previously published studies.

## Supplementary Materials

The following supporting information can be downloaded at: https://www.mdpi.com/article/10.3390/jcm15155792/s1, Additional File S1: Results of the traditional pairwise meta-analysis comparing formulas for the percentage of eyes with refractive prediction error ≤ 0.25 D; Additional File S2: Results of the traditional pairwise meta-analysis comparing formulas for the percentage of eyes with refractive prediction error ≤ 0.50 D; Additional File S3: Results of the traditional pairwise meta-analysis comparing formulas for the percentage of eyes with refractive prediction error ≤ 1.00 D; Additional File S4: Results of the pairwise network meta-analysis comparing formulas for the percentage of eyes with refractive prediction errors ≤ 0.25 D; Additional File S5: Results of the pairwise network meta-analysis comparing formulas for the percentage of eyes with refractive prediction errors ≤ 0.50 D; Additional File S6: Results of the pairwise network meta-analysis comparing formulas for the percentage of eyes with refractive prediction errors ≤ 1.00 D; Additional File S7: Node-split analysis of inconsistency for the percentage of eyes with a refractive prediction error ≤ 0.25 D; Additional File S8: Node-split analysis of inconsistency for the percentage of eyes with a refractive prediction error ≤ 0.50 D; Additional File S9: Node-split analysis of inconsistency for the percentage of eyes with a refractive prediction error ≤ 1.00 D; Additional File S10: Exact SUCRA values and Probability of Ranking First for each formula (Percentage of eyes with a refractive prediction error ≤0.25 D); Additional File S11: Exact SUCRA values and Probability of Ranking First for each formula (Percentage of eyes with a refractive prediction error ≤0.50 D); Additional File S12: Exact SUCRA values and Probability of Ranking First for each formula (Percentage of eyes with a refractive prediction error ≤1.00 D); Additional File S13: Comparison-adjusted funnel plots evaluating the percentage of eyes with a refractive prediction error <0.25 D (A), <0.50 D (B) and <1.00 D (C); Additional File S14: Results of the application of the modified QUADAS-2 tool to the included studies; Additional File S15: Reported MAE and MedAE values for the IOL power calculation formulas evaluated in the included studies; PRISMA Checklist.

## Abbreviations

ACD	Anterior Chamber Depth
AI	artificial intelligence
AL	axial length
CCT	central corneal thickness
D	diopters
ELP	effective lens position
Holladay 1_modified W-K_	Holladay 1 with the modified Wang–Koch AL adjustment
Holladay 1_original W-K_	Holladay 1 with the original Wang–Koch AL adjustment
IOL	intraocular lens
K	Keratometry
LT	Lens Thickness
MAE	mean absolute error
ME	mean error
MedAE	median absolute error
NMA	network meta-analysis
OR	odds ratio
PE	predictive error
PRISMA	Preferred Reporting Items for Systematic Reviews and Meta-Analyses
QUADAS-2	Quality Assessment of Diagnostic Accuracy Studies
RLE	refractive lens exchange
RMSAE	root mean square absolute error
SRK/T_modified W-K_	SRK/T with the modified Wang–Koch AL adjustment
SRK/T_original W-K_	SRK/T with the original Wang–Koch AL adjustment
SUCRA	surface under the cumulative ranking curve
