# Supplementary material for: Evaluation of Intraocular Lens Power Calculation Formulas for Highly Myopic Eyes: A Systematic Review and Network Meta-Analysis
Source: J Clin Med. 2026 Jul 24;15(15):5792. doi: 10.3390/jcm15155792 (PMC13467532; doi:10.3390/jcm15155792)
Supplement: Supplementary file 1 [file jcm-15-05792-s001.zip › Additional File S1.pdf]

Additional File S1. Results of the traditional pairwise meta-analysis comparing formulas for the percentage of eyes with refractive prediction error ≤ 0.25 D.

Formula comparison	Number of studies	OR [CI 95%]	I^2 (%)
Barrett Universal II vs EVO	6	0.956 [0.818, 1.116]	0.00
Barrett Universal II vs Haigis	8	1.082 [0.922, 1.271]	0.00
Barrett Universal II vs Hill RBF 2.0	4	1.014 [0.841, 1.224]	0.00
Barrett Universal II vs Hill RBF 3.0	2	0.816 [0.617, 1.081]	6.00
Barrett Universal II vs Hoffer Q	2	1.449 [0.934, 2.249]	0.00
Barrett Universal II vs Hoffer QST	2	0.937 [0.684, 1.285]	0.00
Barrett Universal II vs Holladay 1	6	1.248 [1.000, 1.558]	0.00
Barrett Universal II vs Holladay $1_{\text{modified W-K}}$	5	1.025 [0.859, 1.224]	0.00
Barrett Universal II vs Holladay $1_{\text{original W-K}}$	3	0.955 [0.787, 1.160]	0.00
Barrett Universal II vs Kane	5	0.901 [0.765, 1.062]	0.00
Barrett Universal II vs LSF	2	1.496 [0.763, 2.933]	62.88
Barrett Universal II vs Pearl-DGS	2	0.962 [0.721, 1.283]	0.00
Barrett Universal II vs SRK/T	8	1.111 [0.927, 1.330]	0.00
Barrett Universal II vs $\text{SRK/T}_{\text{modified W-K}}$	5	1.196 [0.997, 1.436]	0.00
Barrett Universal II vs SRKT (OWK)	3	1.111 [0.911, 1.356]	0.00
EVO vs Haigis	4	1.000 [0.820, 1.220]	0.00
EVO vs Hill RBF 2.0	1	0.961 [0.735, 1.256]	N/A
EVO vs Hill RBF 3.0	2	0.950 [0.723, 1.248]	0.00
EVO vs Hoffer Q	0	N/A	N/A
EVO vs Hoffer QST	2	1.160 [0.856, 1.572]	0.00
EVO vs Holladay 1	3	1.269 [0.928, 1.735]	0.00
EVO vs Holladay $1_{\text{modified W-K}}$	3	0.978 [0.797, 1.201]	0.00
EVO vs Holladay $1_{\text{original W-K}}$	2	0.902 [0.726, 1.121]	0.00
EVO vs Kane	4	0.932 [0.787, 1.104]	0.00
EVO vs LSF	1	1.210 [0.811, 1.805]	N/A
EVO vs Pearl-DGS	2	1.120 [0.845, 1.483]	0.00
EVO vs SRK/T	4	1.109 [0.874, 1.407]	0.00
EVO vs $\text{SRK/T}_{\text{modified W-K}}$	3	1.167 [0.944, 1.442]	0.00
EVO vs $\text{SRK/T}_{\text{original W-K}}$	2	1.047 [0.838, 1.308]	0.00
Haigis vs Hill RBF 2.0	4	0.898 [0.741, 1.088]	0.00
Haigis vs Hill RBF 3.0	1	0.971 [0.547, 1.725]	N/A
Haigis vs Hoffer Q	2	1.189 [0.827, 1.708]	0.00
Haigis vs Hoffer QST	1	1.217 [0.552, 2.683]	N/A
Haigis vs Holladay 1	5	1.168 [0.915, 1.491]	0.00

Haigis vs Holladay $1_{\text{modified W-K}}$	4	0.898 [0.734, 1.097]	0.00
Haigis vs Holladay $1_{\text{original W-K}}$	2	0.897 [0.715, 1.125]	0.00
Haigis vs Kane	4	0.840 [0.693, 1.017]	0.00
Haigis vs LSF	1	1.546 [0.712, 3.357]	N/A
Haigis vs Pearl-DGS	1	0.944 [0.533, 1.672]	N/A
Haigis vs SRK/T	7	1.025 [0.844, 1.245]	0.00
Haigis vs $\text{SRK/T}_{\text{modified W-K}}$	4	1.056 [0.859, 1.298]	0.00
Haigis vs $\text{SRK/T}_{\text{original W-K}}$	2	1.079 [0.854, 1.364]	0.00
Hill RBF 2.0 vs Hill RBF 3.0	0	N/A	N/A
Hill RBF 2.0 vs Hoffer Q	1	1.551 [1.004, 2.397]	0.00
Hill RBF 2.0 vs Hoffer QST	0	N/A	N/A
Hill RBF 2.0 vs Holladay 1	2	1.261 [0.922, 1.724]	0.00
Hill RBF 2.0 vs Holladay 1 MWK	3	0.973 [0.787, 1.203]	0.00
Hill RBF 2.0 vs Holladay $1_{\text{original W-K}}$	2	0.913 [0.728, 1.144]	0.00
Hill RBF 2.0 vs Kane	2	0.892 [0.703, 1.130]	0.00
Hill RBF 2.0 vs LSF	1	2.533 [1.228, 5.222]	N/A
Hill RBF 2.0 vs Pearl-DGS	0	N/A	N/A
Hill RBF 2.0 vs SRK/T	3	1.177 [0.894, 1.549]	0.00
Hill RBF 2.0 vs $\text{SRK/T}_{\text{modified W-K}}$	3	1.139 [0.916, 1.417]	0.00
Hill RBF 2.0 vs SRK/T (0WK)	2	1.099 [0.870, 1.387]	0.00
Hill RBF 3.0 vs Hoffer Q	0	N/A	N/A
Hill RBF 3.0 vs Hoffer QST	1	1.305 [0.946, 1.802]	N/A
Hill RBF 3.0 vs Holladay 1	1	1.296 [0.712, 2.360]	N/A
Hill RBF 3.0 vs Holladay $1_{\text{modified W-K}}$	1	0.875 [0.500, 1.530]	N//A
Hill RBF 3.0 vs Holladay $1_{\text{original W-K}}$	0	N/A	N/A
Hill RBF 3.0 vs Kane	2	1.082 [0.823, 1.424]	0.00
Hill RBF 3.0 vs LSF	0	N/A	N/A
Hill RBF 3.0 vs Pearl-DGS	2	1.178 [0.892, 1.557]	0.00
Hill RBF 3.0 vs SRK/T	1	1.061 [0.596, 1.889]	N/A
Hill RBF 3.0 vs $\text{SRK/T}_{\text{modified W-K}}$	1	1.061 [0.596, 1.889]	N/A
Hill RBF 3.0 vs $\text{SRK/T}_{\text{original W-K}}$	0	N/A	N/A
Hoffer Q vs Hoffer QST	0	N/A	N/A
Hoffer Q vs Holladay 1	2	0.993 [0.684, 1.443]	0.00
Hoffer Q vs Holladay $1_{\text{modified W-K}}$	0	N/A	N/A

Hoffer Q vs Holladay $1_{original\ W\text{-}K}$	0	N/A	N/A
Hoffer Q vs Kane	0	N/A	N/A
Hoffer Q vs LSF	0	N/A	N/A
Hoffer Q vs Pearl-DGS	0	N/A	N/A
Hoffer Q vs SRK/T	2	0.887 [0.615, 1.279]	0.00
Hoffer Q vs $SRK/T_{modified\ W\text{-}K}$	0	N/A	N/A
Hoffer Q vs $SRK/T_{original\ W\text{-}K}$	0	N/A	N/A
Hoffer QST vs Holladay 1	1	0.920 [0.410, 2.062]	N/A
Hoffer QST vs Holladay $1_{modified\ W\text{-}K}$	0	N/A	N/A
Hoffer QST vs Holladay $1_{original\ W\text{-}K}$	0	N/A	N/A
Hoffer QST vs Kane	1	0.880 [0.633, 1.223]	N/A
Hoffer QST vs LSF	0	N/A	N/A
Hoffer QST vs Pearl-DGS	1	0.959 [0.687, 1.340]	N/A
Hoffer QST vs SRK/T	1	0.767 [0.351, 1.674]	N/A
Hoffer QST vs $SRK/T_{modified\ W\text{-}K}$	0	N/A	N/A
Hoffer QST vs $SRK/T_{original\ W\text{-}K}$	0	N/A	N/A
Holladay 1 vs Holladay $1_{modified\ W\text{-}K}$	3	0.845 [0.642, 1.114]	0.00
Holladay 1 vs Holladay $1_{original\ W\text{-}K}$	2	0.780 [0.575, 1.058]	0.00
Holladay 1 vs Kane	1	0.692 [0.384, 1.248]	N/A
Holladay 1 vs LSF	1	0.871 [0.570, 1.331]	N/A
Holladay 1 vs Pearl-DGS	1	0.750 [0.413, 1.362]	N/A
Holladay 1 vs SRK/T	6	0.879 [0.711, 1.087]	0.00
Holladay 1 vs $SRK/T_{modified\ W\text{-}K}$	3	0.977 [0.737, 1.296]	0.00
Holladay 1 vs $SRK/T_{original\ W\text{-}K}$	2	0.861 [0.632, 1.174]	0.00
Holladay $1_{modified\ W\text{-}K}$ vs Holladay $1_{original\ W\text{-}K}$	3	0.918 [0.755, 1.115]	0.00
Holladay $1_{modified\ W\text{-}K}$ vs Kane	3	0.938 [0.756, 1.165]	0.00
Holladay $1_{modified\ W\text{-}K}$ vs LSF	2	1.414 [0.626, 3.192]	73.75
Holladay $1_{modified\ W\text{-}K}$ vs Pearl-DGS	1	1.111 [0.637, 1.938]	N/A
Holladay $1_{modified\ W\text{-}K}$ vs SRK/T	4	1.064 [0.831, 1.362]	0.00
Holladay $1_{modified\ W\text{-}K}$ vs $SRK/T_{modified\ W\text{-}K}$	5	1.167 [0.972, 1.402]	0.00
Holladay $1_{modified\ W\text{-}K}$ vs $SRK/T_{original\ W\text{-}K}$	3	1.067 [0.873, 1.304]	0.00
Holladay $1_{original\ W\text{-}K}$ vs Kane	1	0.966 [0.749, 1.246]	N/A
Holladay $1_{original\ W\text{-}K}$ vs LSF	1	1.226 [0.822, 1.827]	N/A

Holladay $1_{original\ W\text{-}K}$ vs Pearl-DGS	0	N/A	N/A
Holladay $1_{original\ W\text{-}K}$ vs SRK/T	2	1.146 [0.851, 1.544]	0.00
Holladay $1_{original\ W\text{-}K}$ vs SRK/$T_{modified\ W\text{-}K}$	3	1.288 [1.053, 1.576]	0.00
Holladay $1_{original\ W\text{-}K}$ vs SRK/$T_{original\ W\text{-}K}$	3	1.163 [0.955, 1.418]	0.00
Kane vs LSF	1	2.513 [1.218, 5.185]	N/A
Kane vs Pearl-DGS	2	1.089 [0.821, 1.444]	0.00
Kane vs SRK/T	3	1.219 [0.919, 1.616]	0.00
Kane vs SRK/$T_{modified\ W\text{-}K}$	3	1.259 [1.007, 1.574]	0.00
Kane vs SRK/$T_{original\ W\text{-}K}$	1	1.255 [0.964, 1.635]	N/A
LSF vs Pearl-DGS	0	N/A	N/A
LSF vs SRK/T	2	0.794 [0.440, 1.434]	50.86
LSF vs SRK/$T_{modified\ W\text{-}K}$	2	0.745 [0.292, 1.901]	79.76
LSF vs SRK/$T_{original\ W\text{-}K}$	1	0.861 [0.576, 1.288]	N/A
Pearl-DGS vs SRK/T	1	1.091 [0.614, 1.937]	N/A
Pearl-DGS vs SRK/$T_{modified\ W\text{-}K}$	1	1.091 [0.614, 1.937]	N/A
Pearl-DGS vs SRK/$T_{original\ W\text{-}K}$	0	N/A	N/A
SRK/T vs SRK/$T_{modified\ W\text{-}K}$	4	1.060 [0.824, 1.364]	0.00
SRK/T vs SRK/$T_{original\ W\text{-}K}$	2	0.964 [0.711, 1.305]	0.00
SRK/$T_{modified\ W\text{-}K}$ vs SRK/$T_{original\ W\text{-}K}$	3	0.903 [0.734, 1.110]	0.00

OR: odds ratio; CI: confidence interval; I^2: I-squared heterogeneity statistic; N/A: not applicable
