# Supplementary material for: Evaluation of Intraocular Lens Power Calculation Formulas for Highly Myopic Eyes: A Systematic Review and Network Meta-Analysis
Source: J Clin Med. 2026 Jul 24;15(15):5792. doi: 10.3390/jcm15155792 (PMC13467532; doi:10.3390/jcm15155792)
Supplement: Supplementary file 1 [file jcm-15-05792-s001.zip › Additional File S10.pdf]

Additional File S10. Exact SUCRA values and Probability of Ranking First for each formula (Percentage of eyes with a refractive prediction error ≤0.25 D)

**Formula**	**SUCRA (%)**	**Probability of best (%)**	**Mean Rank**
Hill RBF 3.0	95.3	67.4	1.7
Kane	89.9	16.7	2.5
Holladay $1_{\text{original W-K}}$	85	11.7	3.3
Hill RBF 2.0	72.8	2.3	5.1
EVO	69.5	0.4	5.6
Holladay $1_{\text{modified W-K}}$	65.3	0.3	6.2
Barrett Universal II	63.2	0.0	6.5
Pearl-DGS	53.5	0.9	8
Haigis	43.6	0.0	9.5
$\text{SRK/T}_{\text{original W-K}}$	39.9	0.0	10.0
Hoffer QST	37.2	0.2	10.4
SRK/T	33.3	0.0	11.0
$\text{SRK/T}_{\text{modified W-K}}$	22.5	0.0	12.6
LSF	11.5	0.0	14.3
Holladay 1	10.7	0.0	14.4
Hoffer Q	6.9	0.0	15.0
