# Supplementary material for: Evaluation of Intraocular Lens Power Calculation Formulas for Highly Myopic Eyes: A Systematic Review and Network Meta-Analysis
Source: J Clin Med. 2026 Jul 24;15(15):5792. doi: 10.3390/jcm15155792 (PMC13467532; doi:10.3390/jcm15155792)
Supplement: Supplementary file 1 [file jcm-15-05792-s001.zip › Additional File S11.pdf]

Additional File S11. Exact SUCRA values and Probability of Ranking First for each formula (Percentage of eyes with a refractive prediction error ≤0.50 D)

**Formula**	**SUCRA (%)**	**Probability of best (%)**	**Mean Rank**
Kane	90.5	33.1	2.4
Holladay $1_{\text{original W-K}}$	90.3	38.7	2.5
EVO	80.1	8	4
Hill RBF 2.0	71.4	4.6	5.3
Hill RBF 3.0	67.3	10.5	5.9
Holladay $1_{\text{modified W-K}}$	66.6	1.6	6
Barrett Universal II	60.8	0.2	6.9
$\text{SRK/T}_{\text{original W-K}}$	50.9	0.2	8.4
Hoffer QST	47.3	1.6	8.9
Pearl-DGS	45.2	1.5	9.2
$\text{SRK/T}_{\text{modified W-K}}$	43.7	0.1	9.4
Haigis	42.0	0.0	9.7
SRK/T	21.8	0.0	12.7
Holladay 1	9.4	0.0	14.6
LSF	9.1	0.0	14.6
Hoffer Q	3.4	0.0	15.5
