# Supplementary material for: Evaluation of Intraocular Lens Power Calculation Formulas for Highly Myopic Eyes: A Systematic Review and Network Meta-Analysis
Source: J Clin Med. 2026 Jul 24;15(15):5792. doi: 10.3390/jcm15155792 (PMC13467532; doi:10.3390/jcm15155792)
Supplement: Supplementary file 1 [file jcm-15-05792-s001.zip › Additional File S12.pdf]

Additional File S12. Exact SUCRA values and Probability of Ranking First for each formula (Percentage of eyes with a refractive prediction error ≤1.00 D)

**Formula**	**SUCRA (%)**	**Probability of best (%)**	**Mean Rank**
Holladay $1_{\text{original W-K}}$	88.7	35.1	2.7
Kane	85.8	19.9	3.1
Barrett Universal II	76.5	3.3	4.5
Hill RBF 2.0	76.3	7.2	4.6
Pearl-DGS	71.1	19.8	5.3
Hill RBF 3.0	66.5	13.3	6
EVO	64.4	0.8	6.3
Holladay $1_{\text{modified W-K}}$	56.9	0.2	7.5
SRK/$T_{\text{modified W-K}}$	45.3	0.0	9.2
SRK/$T_{\text{original W-K}}$	42.2	0.0	9.7
Haigis	40.8	0.0	9.9
Hoffer QST	36.8	0.3	10.5
SRK/T	21.2	0.0	12.8
LSF	16.3	0.0	13.6
Holladay 1	9.2	0.0	14.6
Hoffer Q	2.1	0.0	15.7
