# Supplementary material for: Evaluation of Intraocular Lens Power Calculation Formulas for Highly Myopic Eyes: A Systematic Review and Network Meta-Analysis
Source: J Clin Med. 2026 Jul 24;15(15):5792. doi: 10.3390/jcm15155792 (PMC13467532; doi:10.3390/jcm15155792)
Supplement: Supplementary file 1 [file jcm-15-05792-s001.zip › Additional File S13.pdf]

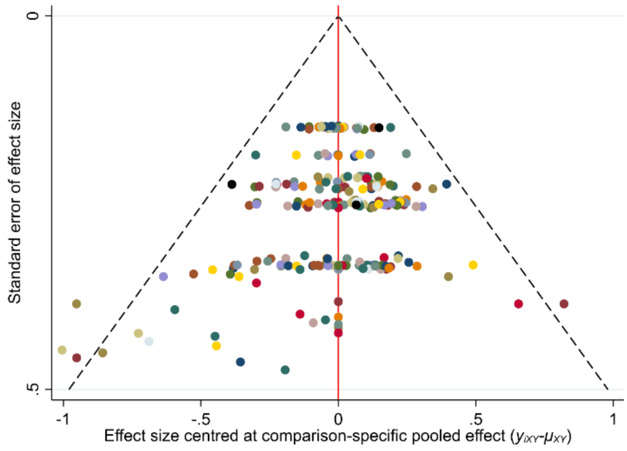

A

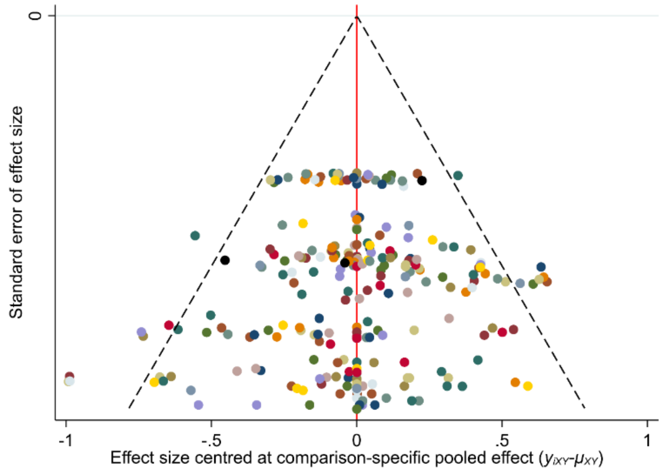

B

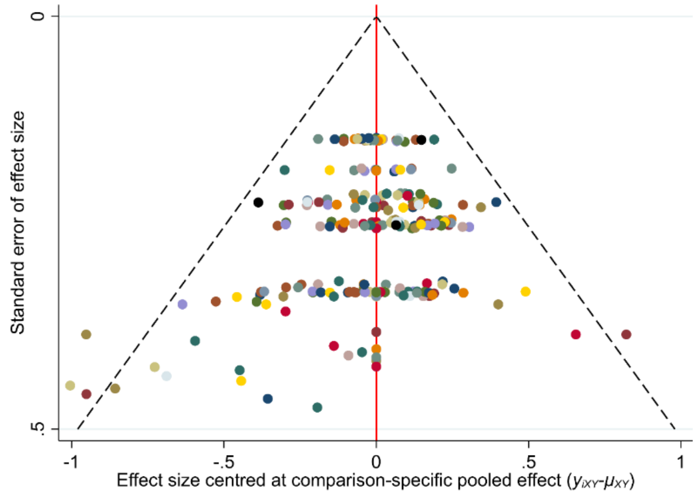

C

Additional File S13. Comparison-adjusted funnel plots evaluating the percentage of eyes with a refractive prediction error <0.25 D (A), <0.50 D (B) and <1.00 D (C).
