# Supplementary material for: Evaluation of Intraocular Lens Power Calculation Formulas for Highly Myopic Eyes: A Systematic Review and Network Meta-Analysis
Source: J Clin Med. 2026 Jul 24;15(15):5792. doi: 10.3390/jcm15155792 (PMC13467532; doi:10.3390/jcm15155792)
Supplement: Supplementary file 1 [file jcm-15-05792-s001.zip › Additional File S15.pdf]

Additional File S15. Reported MAE and MedAE values for the IOL power calculation formulas evaluated in the included studies.

Study	Formula	MAE	SD	MedAE	IQR
Vilaltella M, et al.[7]	Kane	0.329	0.274	0.233	0.365
Vilaltella M, et al.[7]	EVO 2.0	0.331	0.266	0.252	0.354
Vilaltella M, et al.[7]	Barrett Universal II	0.317	0.249	0.246	0.364
Vilaltella M, et al.[7]	Hill RBF 3.0	0.349	0.282	0.255	0.383
Vilaltella M, et al.[7]	Pearl-DGS	0.324	0.276	0.252	0.337
Vilaltella M, et al.[7]	SRK/T	0.414	0.317	0.291	0.401
Vilaltella M, et al.[7]	Haigis	0.358	0.307	0.275	0.447
Vilaltella M, et al.[7]	Holladay 1	0.408	0.303	0.350	0.429
Vilaltella M, et al.[7]	Holladay 2	0.378	0.328	0.277	0.390
Vilaltella M, et al.[7]	SRK/T (MWK)	0.360	0.307	0.300	0.404
Vilaltella M, et al.[7]	Holladay 1 (MWK)	0.314	0.289	0.215	0.367
Vilaltella M, et al.[7]	Holladay 2 (non-linear WK)	0.341	0.292	0.261	0.334
Liu J, et al.[19]	Barrett Universal II	0.32	N/A	0.27	N/A
Liu J, et al.[19]	Hill RBF 2.0	0.37	N/A	0.33	N/A
Liu J, et al.[19]	SRK/T	0.49	N/A	0.34	N/A
Liu J, et al.[19]	Haigis	0.41	N/A	0.36	N/A
Liu J, et al.[19]	Holladay 1	0.45	N/A	0.40	N/A
Liu J, et al.[19]	SRK/T (MWK)	0.47	N/A	0.43	N/A
Liu J, et al.[19]	SRK/T (OWK)	0.46	N/A	0.40	N/A
Liu J, et al.[19]	Holladay 1 (MWK)	0.39	N/A	0.38	N/A
Liu J, et al.[19]	Holladay 1 (OWK)	0.37	N/A	0.34	N/A
Zhang J, et al.[37]	EVO 2.0	0.35	N/A	0.27	N/A
Zhang J, et al.[37]	Barrett Universal II	0.38	N/A	0.28	N/A

Zhang J, et al.[37]	LSF	0.43	N/A	0.32	N/A
Zhang J, et al.[37]	SRK/T	0.43	N/A	0.35	N/A
Zhang J, et al.[37]	Holladay 1	0.48	N/A	0.40	N/A
Zhang J, et al.[37]	SRK/T (MWK)	0.42	N/A	0.36	N/A
Zhang J, et al.[37]	SRK/T (OWK)	0.38	N/A	0.29	N/A
Zhang J, et al.[37]	Holladay 1 (MWK)	0.40	N/A	0.33	N/A
Zhang J, et al.[37]	Holladay 1 (OWK)	0.35	N/A	0.27	N/A
Zhang J, et al.[37]	Holladay 1 (non-linear AL)	0.36	N/A	0.28	N/A
Zhang J, et al.[37]	Holladay 1 (CMAL)	0.42	N/A	0.35	N/A
Zhang J, et al.[37]	EVO (CMAL)	0.36	N/A	0.28	N/A
Zhang J, et al.[37]	BU II (CMAL)	0.38	N/A	0.30	N/A
Zhang J, et al.[37]	LSF (CMAL)	0.40	N/A	0.29	N/A
Zhang J, et al.[37]	SRK/T (CMAL)	0.40	N/A	0.31	N/A
Zhang J, et al.[38]	SRK/T	0.72	0.56	0.64	N/A
Zhang J, et al.[38]	Haigis	0.65	0.51	0.55	N/A
Zhang J, et al.[38]	Hoffer Q	0.99	0.76	0.85	N/A
Zhang J, et al.[38]	Holladay 1	0.89	0.65	0.78	N/A
Cheng H, et al.[17]	Kane	0.34	N/A	0.26	N/A
Cheng H, et al.[17]	EVO	0.40	N/A	0.31	N/A
Cheng H, et al.[17]	Barrett Universal II	0.37	N/A	0.31	N/A
Cheng H, et al.[17]	Hill RBF 2.0	0.38	N/A	0.30	N/A
Cheng H, et al.[17]	Haigis	0.40	N/A	0.34	N/A
Cheng H, et al.[17]	SRK/T (MWK)	0.39	N/A	0.32	N/A
Cheng H, et al.[17]	SRK/T (OWK)	0.39	N/A	0.32	N/A

Cheng H, et al.[17]	Holladay 1 (MWK)	0.34	N/A	0.28	N/A
Cheng H, et al.[17]	Holladay 1 (OWK)	0.34	N/A	0.27	N/A
Whang W, et al.[36]	EVO 2.0	0.59	N/A	0.60	N/A
Whang W, et al.[36]	Barrett Universal II	0.60	N/A	0.61	N/A
Whang W, et al.[36]	Hoffer QST	0.56	N/A	0.55	N/A
Whang W, et al.[36]	SRK/T	0.58	N/A	0.59	N/A
Whang W, et al.[36]	Haigis	0.56	N/A	0.53	N/A
Whang W, et al.[36]	Holladay 1	0.63	N/A	0.51	N/A
Mo E, et al.[35]	Kane	0.42	N/A	0.36	N/A
Mo E, et al.[35]	EVO 2.0	0.32	N/A	0.26	N/A
Mo E, et al.[35]	Barrett Universal II	0.37	N/A	0.31	N/A
Mo E, et al.[35]	Hill RBF 3.0	0.36	N/A	0.31	N/A
Mo E, et al.[35]	Hoffer QST	0.44	N/A	0.39	N/A
Mo E, et al.[35]	Pearl-DGS	0.37	N/A	0.29	N/A
Mo E, et al.[35]	Cooke K6	0.33	N/A	0.27	N/A
Lin L, et al.[5]	Kane	0.318	0.227	0.271	N/A
Lin L, et al.[5]	EVO 2.0	0.314	0.216	0.288	N/A
Lin L, et al.[5]	Barrett Universal II	0.342	0.253	0.297	N/A
Lin L, et al.[5]	SRK/T	0.398	0.301	0.345	N/A
Lin L, et al.[5]	Haigis	0.336	0.247	0.283	N/A
Bernardes J, et al.[4]	Kane	0.42	N/A	0.33	N/A
Bernardes J, et al.[4]	Barrett Universal II	0.43	N/A	0.36	N/A
Bernardes J, et al.[4]	Hill RBF 2.0	0.39	N/A	0.31	N/A
Bernardes J, et al.[4]	LSF	0.66	N/A	0.61	N/A
Bernardes J, et al.[4]	SRK/T	0.53	N/A	0.43	N/A

Bernardes J, et al.[4]	Haigis	0.53	N/A	0.54	N/A
Bernardes J, et al.[4]	SRK/T (MWK)	0.47	N/A	0.37	N/A
Bernardes J, et al.[4]	Holladay 1 (MWK)	0.64	N/A	0.43	N/A
Wan K, et al.[8]	Barrett Universal II	N/A	N/A	0.21	N/A
Wan K, et al.[8]	Hill RBF 2.0	N/A	N/A	0.20	N/A
Wan K, et al.[8]	SRK/T	N/A	N/A	0.27	N/A
Wan K, et al.[8]	Haigis	N/A	N/A	0.28	N/A
Wan K, et al.[8]	Hoffer Q	N/A	N/A	0.33	N/A
Wan K, et al.[8]	Holladay 1	N/A	N/A	0.30	N/A
