# Supplementary material for: Evaluation of Intraocular Lens Power Calculation Formulas for Highly Myopic Eyes: A Systematic Review and Network Meta-Analysis
Source: J Clin Med. 2026 Jul 24;15(15):5792. doi: 10.3390/jcm15155792 (PMC13467532; doi:10.3390/jcm15155792)
Supplement: Supplementary file 1 [file jcm-15-05792-s001.zip › Additional File S2.pdf]

Additional File S2. Results of the traditional pairwise meta-analysis comparing formulas for the percentage of eyes with refractive prediction error ≤ 0.50 D.

**Formula comparison**	**Number of studies**	**OR [CI 95%]**	**I² (%)**
Barrett Universal II vs EVO	6	0.953 [0.837, 1.086]	0.00
Barrett Universal II vs Haigis	8	1.053 [0.921, 1.205]	0.00
Barrett Universal II vs Hill RBF 2.0	4	1.027 [0.876, 1.204]	0.00
Barrett Universal II vs Hill RBF 3.0	2	0.940 [0.738, 1.198]	0.00
Barrett Universal II vs Hoffer Q	2	1.183 [0.817, 1.712]	0.00
Barrett Universal II vs Hoffer QST	2	0.930 [0.720, 1.203]	0.00
Barrett Universal II vs Holladay 1	6	1.171 [0.974, 1.409]	0.00
Barrett Universal II vs Holladay $1_{\text{modified W-K}}$	5	1.025 [0.883, 1.190]	0.00
Barrett Universal II vs Holladay $1_{\text{original W-K}}$	3	0.986 [0.837, 1.162]	0.00
Barrett Universal II vs Kane	5	0.946 [0.823, 1.087]	0.00
Barrett Universal II vs LSF	2	1.212 [0.912, 1.611]	44.74
Barrett Universal II vs Pearl-DGS	2	0.973 [0.763, 1.242]	0.00
Barrett Universal II vs SRK/T	8	1.105 [0.949, 1.287]	0.00
Barrett Universal II vs $\text{SRK/T}_{\text{modified W-K}}$	5	1.063 [0.915, 1.235]	0.00
Barrett Universal II vs SRKT (OWK)	3	1.050 [0.890, 1.239]	0.00
EVO vs Haigis	4	1.007 [0.853, 1.189]	0.00
EVO vs Hill RBF 2.0	1	0.996 [0.796, 1.247]	N/A
EVO vs Hill RBF 3.0	2	1.073 [0.846, 1.361]	0.00
EVO vs Hoffer Q	0	N/A	N/A
EVO vs Hoffer QST	2	1.063 [0.826, 1.368]	0.16
EVO vs Holladay 1	3	1.111 [0.859, 1.436]	16.99
EVO vs Holladay $1_{\text{modified W-K}}$	3	0.978 [0.823, 1.162]	0.00
EVO vs Holladay $1_{\text{original W-K}}$	2	0.957 [0.797, 1.151]	0.00
EVO vs Kane	4	0.994 [0.861, 1.148]	0.00
EVO vs LSF	1	1.161 [0.832, 1.619]	N/A
EVO vs Pearl-DGS	2	1.111 [0.874, 1.411]	0.00
EVO vs SRK/T	4	1.142 [0.934, 1.395]	0.00
EVO vs $\text{SRK/T}_{\text{modified W-K}}$	3	1.027 [0.863, 1.222]	0.00
EVO vs $\text{SRK/T}_{\text{original W-K}}$	2	1.002 [0.833, 1.206]	0.00
Haigis vs Hill RBF 2.0	4	0.948 [0.807, 1.114]	0.00
Haigis vs Hill RBF 3.0	1	1.000 [0.600, 1.666]	N/A
Haigis vs Hoffer Q	2	1.222 [0.906, 1.648]	0.00
Haigis vs Hoffer QST	1	1.114 [0.593, 2.092]	N/A
Haigis vs Holladay 1	5	1.166 [0.952, 1.428]	0.00

Haigis vs Holladay $1_{modified\ W-K}$	4	0.928 [0.784, 1.099]	0.00
Haigis vs Holladay $1_{original\ W-K}$	2	0.928 [0.767, 1.122]	0.00
Haigis vs Kane	4	0.913 [0.776, 1.074]	0.00
Haigis vs LSF	1	1.234 [0.703, 2.167]	N/A
Haigis vs Pearl-DGS	1	0.893 [0.540, 1.475]	N/A
Haigis vs SRK/T	7	1.050 [0.893, 1.234]	0.00
Haigis vs $SRK/T_{modified\ W-K}$	4	0.973 [0.821, 1.153]	0.00
Haigis vs $SRK/T_{original\ W-K}$	2	1.017 [0.839, 1.233]	0.00
Hill RBF 2.0 vs Hill RBF 3.0	0	N/A	N/A
Hill RBF 2.0 vs Hoffer Q	1	1.183 [0.817, 1.712]	N/A
Hill RBF 2.0 vs Hoffer QST	0	N/A	N/A
Hill RBF 2.0 vs Holladay 1	2	1.265 [0.971, 1.648]	0.00
Hill RBF 2.0 vs Holladay 1 MWK	3	0.988 [0.827, 1.180]	0.00
Hill RBF 2.0 vs Holladay $1_{original\ W-K}$	2	0.951 [0.787, 1.149]	0.00
Hill RBF 2.0 vs Kane	2	0.935 [0.764, 1.145]	0.00
Hill RBF 2.0 vs LSF	1	1.653 [0.963, 2.836]	N/A
Hill RBF 2.0 vs Pearl-DGS	0	N/A	N/A
Hill RBF 2.0 vs SRK/T	3	1.129 [0.896, 1.422]	0.00
Hill RBF 2.0 vs $SRK/T_{modified\ W-K}$	3	1.034 [0.865, 1.237]	0.00
Hill RBF 2.0 vs SRK/T (0WK)	2	1.042 [0.860, 1.263]	0.00
Hill RBF 3.0 vs Hoffer Q	0	N/A	N/A
Hill RBF 3.0 vs Hoffer QST	1	1.052 [0.799, 1.384]	N/A
Hill RBF 3.0 vs Holladay 1	1	1.020 [0.612, 1.702]	N/A
Hill RBF 3.0 vs Holladay $1_{modified\ W-K}$	1	0.893 [0.540, 1.475]	N/A
Hill RBF 3.0 vs Holladay $1_{original\ W-K}$	0	N/A	N/A
Hill RBF 3.0 vs Kane	2	0.971 [0.764, 1.233]	0.00
Hill RBF 3.0 vs LSF	0	N/A	N/A
Hill RBF 3.0 vs Pearl-DGS	2	1.035 [0.813, 1.318]	0.00
Hill RBF 3.0 vs SRK/T	1	1.111 [0.662, 1.866]	N/A
Hill RBF 3.0 vs $SRK/T_{modified\ W-K}$	1	0.943 [0.569, 1.565]	N/A
Hill RBF 3.0 vs $SRK/T_{original\ W-K}$	0	N/A	N/A
Hoffer Q vs Hoffer QST	0	N/A	N/A
Hoffer Q vs Holladay 1	2	1.032 [0.757, 1.407]	0.00
Hoffer Q vs Holladay $1_{modified\ W-K}$	0	N/A	N/A

Hoffer Q vs Holladay $1_{original\ W\text{-}K}$	0	N/A	N/A
Hoffer Q vs Kane	0	N/A	N/A
Hoffer Q vs LSF	0	N/A	N/A
Hoffer Q vs Pearl-DGS	0	N/A	N/A
Hoffer Q vs SRK/T	2	0.862 [0.638, 1.165]	0.00
Hoffer Q vs SRK/$T_{modified\ W\text{-}K}$	0	N/A	N/A
Hoffer Q vs SRK/$T_{original\ W\text{-}K}$	0	N/A	N/A
Hoffer QST vs Holladay 1	1	0.898 [0.478, 1.687]	N/A
Hoffer QST vs Holladay $1_{modified\ W\text{-}K}$	0	N/A	N/A
Hoffer QST vs Holladay $1_{original\ W\text{-}K}$	0	N/A	N/A
Hoffer QST vs Kane	1	0.941 [0.715, 1.238]	N/A
Hoffer QST vs LSF	0	N/A	N/A
Hoffer QST vs Pearl-DGS	1	1.029 [0.780, 1.359]	N/A
Hoffer QST vs SRK/T	1	0.957 [0.506, 1.809]	N/A
Hoffer QST vs SRK/$T_{modified\ W\text{-}K}$	0	N/A	N/A
Hoffer QST vs SRK/$T_{original\ W\text{-}K}$	0	N/A	N/A
Holladay 1 vs Holladay $1_{modified\ W\text{-}K}$	3	0.906 [0.721, 1.139]	0.00
Holladay 1 vs Holladay $1_{original\ W\text{-}K}$	2	0.831 [0.645, 1.071]	0.00
Holladay 1 vs Kane	1	0.891 [0.538, 1.476]	N/A
Holladay 1 vs LSF	1	0.929 [0.659, 1.309]	N/A
Holladay 1 vs Pearl-DGS	1	0.875 [0.529, 1.448]	N/A
Holladay 1 vs SRK/T	6	0.937 [0.785, 1.117]	N/A
Holladay 1 vs SRK/$T_{modified\ W\text{-}K}$	3	0.933 [0.742, 1.174]	0.00
Holladay 1 vs SRK/$T_{original\ W\text{-}K}$	2	0.869 [0.674, 1.121]	0.00
Holladay $1_{modified\ W\text{-}K}$ vs Holladay $1_{original\ W\text{-}K}$	3	0.961 [0.816, 1.133]	0.00
Holladay $1_{modified\ W\text{-}K}$ vs Kane	3	1.003 [0.833, 1.208]	0.00
Holladay $1_{modified\ W\text{-}K}$ vs LSF	2	1.141 [0.857, 1.521]	43.74
Holladay $1_{modified\ W\text{-}K}$ vs Pearl-DGS	1	1.000 [0.610, 1.639]	N/A
Holladay $1_{modified\ W\text{-}K}$ vs SRK/T	4	1.071 [0.870, 1.318]	0.00
Holladay $1_{modified\ W\text{-}K}$ vs SRK/$T_{modified\ W\text{-}K}$	5	1.037 [0.892, 1.205]	0.00
Holladay $1_{modified\ W\text{-}K}$ vs SRK/$T_{original\ W\text{-}K}$	3	1.024 [0.867, 1.209]	0.00
Holladay $1_{original\ W\text{-}K}$ vs Kane	1	1.022 [0.820, 1.273]	N/A
Holladay $1_{original\ W\text{-}K}$ vs LSF	1	1.089 [0.779, 1.524]	N/A

Holladay $1_{original\ W\text{-}K}$ vs Pearl-DGS	0	N/A	N/A
Holladay $1_{original\ W\text{-}K}$ vs SRK/T	2	1.154 [0.898, 1.484]	0.00
Holladay $1_{original\ W\text{-}K}$ vs $SRK/T_{modified\ W\text{-}K}$	3	1.095 [0.928, 1.293]	0.00
Holladay $1_{original\ W\text{-}K}$ vs $SRK/T_{original\ W\text{-}K}$	3	1.065 [0.903, 1.256]	0.00
Kane vs LSF	1	1.766 [1.034, 3.017]	N/A
Kane vs Pearl-DGS	2	1.067 [0.839, 1.357]	0.00
Kane vs SRK/T	3	1.151 [0.907, 1.460]	0.00
Kane vs $SRK/T_{modified\ W\text{-}K}$	3	1.041 [0.864, 1.255]	0.00
Kane vs $SRK/T_{original\ W\text{-}K}$	1	1.057 [0.847, 1.320]	N/A
LSF vs Pearl-DGS	0	N/A	N/A
LSF vs SRK/T	2	0.853 [0.542, 1.340]	51.85
LSF vs $SRK/T_{modified\ W\text{-}K}$	2	0.780 [0.460, 1.322]	64.75
LSF vs $SRK/T_{original\ W\text{-}K}$	1	0.896 [0.641, 1.252]	N/A
Pearl-DGS vs SRK/T	1	1.244 [0.747, 2.074]	N/A
Pearl-DGS vs $SRK/T_{modified\ W\text{-}K}$	1	1.057 [0.642, 1.738]	N/A
Pearl-DGS vs $SRK/T_{original\ W\text{-}K}$	0	N/A	N/A
SRK/T vs $SRK/T_{modified\ W\text{-}K}$	4	0.938 [0.762, 1.154]	0.00
SRK/T vs $SRK/T_{original\ W\text{-}K}$	2	0.906 [0.703, 1.167]	0.00
$SRK/T_{modified\ W\text{-}K}$ vs $SRK/T_{original\ W\text{-}K}$	3	0.972 [0.823, 1.149]	0.00

OR: odds ratio; CI: confidence interval; I^2: I-squared heterogeneity statistic; N/A: not applicable
