# Supplementary material for: Evaluation of Intraocular Lens Power Calculation Formulas for Highly Myopic Eyes: A Systematic Review and Network Meta-Analysis
Source: J Clin Med. 2026 Jul 24;15(15):5792. doi: 10.3390/jcm15155792 (PMC13467532; doi:10.3390/jcm15155792)
Supplement: Supplementary file 1 [file jcm-15-05792-s001.zip › Additional File S3.pdf]

Additional File S3. Results of the traditional pairwise meta-analysis comparing formulas for the percentage of eyes with refractive prediction error ≤ 1.00 D.

**Formula comparison**	**Number of studies**	**OR [CI 95%]**	**I²**
Barrett Universal II vs EVO	6	0.999 [0.885, 1.127]	0.00
Barrett Universal II vs Haigis	8	1.026 [0.906, 1.162]	0.00
Barrett Universal II vs Hill RBF 2.0	4	1.009 [0.870, 1.170]	0.00
Barrett Universal II vs Hill RBF 3.0	2	1.003 [0.801, 1.256]	0.00
Barrett Universal II vs Hoffer Q	2	1.042 [0.733, 1.480]	0.00
Barrett Universal II vs Hoffer QST	2	1.011 [0.802, 1.274]	0.00
Barrett Universal II vs Holladay 1	6	1.040 [0.879, 1.232]	0.00
Barrett Universal II vs Holladay $1_{\text{modified W-K}}$	5	1.015 [0.884, 1.165]	0.00
Barrett Universal II vs Holladay $1_{\text{original W-K}}$	3	0.990 [0.849, 1.153]	0.00
Barrett Universal II vs Kane	5	1.003 [0.881, 1.141]	0.00
Barrett Universal II vs LSF	2	1.073 [0.830, 1.386]	0.00
Barrett Universal II vs Pearl-DGS	2	1.000 [0.799, 1.252]	0.00
Barrett Universal II vs SRK/T	8	1.046 [0.910, 1.203]	0.00
Barrett Universal II vs $\text{SRK/T}_{\text{modified W-K}}$	5	1.024 [0.892, 1.176]	0.00
Barrett Universal II vs SRKT (OWK)	3	1.019 [0.874, 1.188]	0.00
EVO vs Haigis	4	1.001 [0.859, 1.166]	0.00
EVO vs Hill RBF 2.0	1	0.983 [0.799, 1.209]	N/A
EVO vs Hill RBF 3.0	2	1.013 [0.810, 1.268]	0.00
EVO vs Hoffer Q	0	N/A	N/A
EVO vs Hoffer QST	2	1.033 [0.820, 1.301]	0.00
EVO vs Holladay 1	3	1.076 [0.853, 1.358]	0.00
EVO vs Holladay $1_{\text{modified W-K}}$	3	0.990 [0.842, 1.163]	0.00
EVO vs Holladay $1_{\text{original W-K}}$	2	0.981 [0.826, 1.164]	0.00
EVO vs Kane	4	0.994 [0.868, 1.138]	0.00
EVO vs LSF	1	1.053 [0.772, 1.438]	N/A
EVO vs Pearl-DGS	2	1.010 [0.807, 1.264]	0.00
EVO vs SRK/T	4	1.044 [0.869, 1.253]	0.00
EVO vs $\text{SRK/T}_{\text{modified W-K}}$	3	1.007 [0.857, 1.183]	0.00
EVO vs $\text{SRK/T}_{\text{original W-K}}$	2	1.004 [0.845, 1.193]	0.00
Haigis vs Hill RBF 2.0	4	0.976 [0.841, 1.133]	0.00
Haigis vs Hill RBF 3.0	1	0.986 [0.618, 1.571]	N/A
Haigis vs Hoffer Q	2	1.141 [0.872, 1.495]	0.00
Haigis vs Hoffer QST	1	0.941 [0.555, 1.596]	N/A
Haigis vs Holladay 1	5	1.075 [0.896, 1.289]	0.00

Haigis vs Holladay $1_{\text{modified W-K}}$	4	0.984 [0.843, 1.149]	0.00
Haigis vs Holladay $1_{\text{original W-K}}$	2	0.979 [0.821, 1.167]	0.00
Haigis vs Kane	4	0.975 [0.840, 1.133]	0.00
Haigis vs LSF	1	0.960 [0.606, 1.522]	N/A
Haigis vs Pearl-DGS	1	1.000 [0.627, 1.595]	N/A
Haigis vs SRK/T	7	1.019 [0.880, 1.179]	0.00
Haigis vs $\text{SRK/T}_{\text{modified W-K}}$	4	0.992 [0.849, 1.158]	0.00
Haigis vs $\text{SRK/T}_{\text{original W-K}}$	2	1.013 [0.849, 1.209]	0.00
Hill RBF 2.0 vs Hill RBF 3.0	0	N/A	N/A
Hill RBF 2.0 vs Hoffer Q	1	1.025 [0.721, 1.457]	N/A
Hill RBF 2.0 vs Hoffer QST	0	N/A	N/A
Hill RBF 2.0 vs Holladay 1	2	1.023 [0.801, 1.306]	0.00
Hill RBF 2.0 vs Holladay 1 MWK	3	1.013 [0.860, 1.194]	0.00
Hill RBF 2.0 vs Holladay $1_{\text{original W-K}}$	2	0.984 [0.826, 1.174]	0.00
Hill RBF 2.0 vs Kane	2	0.996 [0.826, 1.200]	0.00
Hill RBF 2.0 vs LSF	1	1.172 [0.748, 1.834]	N/A
Hill RBF 2.0 vs Pearl-DGS	0	N/A	N/A
Hill RBF 2.0 vs SRK/T	3	1.070 [0.863, 1.326]	0.00
Hill RBF 2.0 vs $\text{SRK/T}_{\text{modified W-K}}$	3	1.022 [0.868, 1.204]	0.00
Hill RBF 2.0 vs SRK/T (0WK)	2	1.019 [0.854, 1.216]	0.00
Hill RBF 3.0 vs Hoffer Q	0	N/A	N/A
Hill RBF 3.0 vs Hoffer QST	1	1.022 [0.790, 1.323]	N/A
Hill RBF 3.0 vs Holladay 1	1	1.014 [0.637, 1.617]	N/A
Hill RBF 3.0 vs Holladay $1_{\text{modified W-K}}$	1	1.014 [0.637, 1.617]	N/A
Hill RBF 3.0 vs Holladay $1_{\text{original W-K}}$	0	N/A	N/A
Hill RBF 3.0 vs Kane	2	0.997 [0.796, 1.248]	0.00
Hill RBF 3.0 vs LSF	0	N/A	N/A
Hill RBF 3.0 vs Pearl-DGS	2	0.997 [0.796, 1.248]	0.00
Hill RBF 3.0 vs SRK/T	1	1.029 [0.645, 1.642]	N/A
Hill RBF 3.0 vs $\text{SRK/T}_{\text{modified W-K}}$	1	1.014 [0.637, 1.617]	N/A
Hill RBF 3.0 vs $\text{SRK/T}_{\text{original W-K}}$	0	N/A	N/A
Hoffer Q vs Hoffer QST	0	N/A	N/A
Hoffer Q vs Holladay 1	2	0.994 [0.755, 1.308]	0.00
Hoffer Q vs Holladay $1_{\text{modified W-K}}$	0	N/A	N/A

Hoffer Q vs Holladay $1_{original\ W\text{-}K}$	0	N/A	N/A
Hoffer Q vs Kane	0	N/A	N/A
Hoffer Q vs LSF	0	N/A	N/A
Hoffer Q vs Pearl-DGS	0	N/A	N/A
Hoffer Q vs SRK/T	2	0.888 [0.678, 1.164]	0.00
Hoffer Q vs $SRK/T_{modified\ W\text{-}K}$	0	N/A	N/A
Hoffer Q vs $SRK/T_{original\ W\text{-}K}$	0	N/A	N/A
Hoffer QST vs Holladay 1	1	1.214 [0.709, 2.081]	N/A
Hoffer QST vs Holladay $1_{modified\ W\text{-}K}$	0	N/A	N/A
Hoffer QST vs Holladay $1_{original\ W\text{-}K}$	0	N/A	N/A
Hoffer QST vs Kane	1	0.974 [0.753, 1.260]	N/A
Hoffer QST vs LSF	0	N/A	N/A
Hoffer QST vs Pearl-DGS	1	0.970 [0.750, 1.254]	N/A
Hoffer QST vs SRK/T	1	1.076 [0.634, 1.826]	N/A
Hoffer QST vs $SRK/T_{modified\ W\text{-}K}$	0	N/A	N/A
Hoffer QST vs $SRK/T_{original\ W\text{-}K}$	0	N/A	N/A
Holladay 1 vs Holladay $1_{modified\ W\text{-}K}$	3	0.978 [0.796, 1.202]	0.00
Holladay 1 vs Holladay $1_{original\ W\text{-}K}$	2	0.961 [0.764, 1.208]	0.00
Holladay 1 vs Kane	1	0.986 [0.618, 1.571]	N/A
Holladay 1 vs LSF	1	1.007 [0.736, 1.376]	N/A
Holladay 1 vs Pearl-DGS	1	1.000 [0.627, 1.595]	N/A
Holladay 1 vs SRK/T	6	0.969 [0.828, 1.134]	0.00
Holladay 1 vs $SRK/T_{modified\ W\text{-}K}$	3	0.999 [0.813, 1.228]	0.00
Holladay 1 vs $SRK/T_{original\ W\text{-}K}$	2	0.991 [0.787, 1.247]	0.00
Holladay $1_{modified\ W\text{-}K}$ vs Holladay $1_{original\ W\text{-}K}$	3	0.993 [0.852, 1.157]	0.00
Holladay $1_{modified\ W\text{-}K}$ vs Kane	3	0.980 [0.824, 1.165]	0.00
Holladay $1_{modified\ W\text{-}K}$ vs LSF	2	1.029 [0.796, 1.331]	0.00
Holladay $1_{modified\ W\text{-}K}$ vs Pearl-DGS	1	1.000 [0.627, 1.595]	N/A
Holladay $1_{modified\ W\text{-}K}$ vs SRK/T	4	1.030 [0.853, 1.243]	0.00
Holladay $1_{modified\ W\text{-}K}$ vs $SRK/T_{modified\ W\text{-}K}$	5	1.009 [0.878, 1.159]	0.00
Holladay $1_{modified\ W\text{-}K}$ vs $SRK/T_{original\ W\text{-}K}$	3	1.022 [0.877, 1.192]	0.00
Holladay $1_{original\ W\text{-}K}$ vs Kane	1	1.006 [0.819, 1.235]	N/A
Holladay $1_{original\ W\text{-}K}$ vs LSF	1	1.047 [0.767, 1.429]	N/A

Holladay $1_{original\ W\text{-}K}$ vs Pearl-DGS	0	N/A	N/A
Holladay $1_{original\ W\text{-}K}$ vs SRK/T	2	1.075 [0.854, 1.353]	0.00
Holladay $1_{original\ W\text{-}K}$ vs $SRK/T_{modified\ W\text{-}K}$	3	1.032 [0.885, 1.203]	0.00
Holladay $1_{original\ W\text{-}K}$ vs $SRK/T_{original\ W\text{-}K}$	3	1.030 [0.884, 1.200]	0.00
Kane vs LSF	1	1.153 [0.736, 1.807]	N/A
Kane vs Pearl-DGS	2	1.000 [0.799, 1.252]	0.00
Kane vs SRK/T	3	1.038 [0.834, 1.292]	0.00
Kane vs $SRK/T_{modified\ W\text{-}K}$	3	1.020 [0.857, 1.213]	0.00
Kane vs $SRK/T_{original\ W\text{-}K}$	1	1.023 [0.832, 1.257]	N/A
LSF vs Pearl-DGS	0	N/A	N/A
LSF vs SRK/T	2	0.962 [0.744, 1.244]	0.00
LSF vs $SRK/T_{modified\ W\text{-}K}$	2	0.945 [0.731, 1.221]	0.00
LSF vs $SRK/T_{original\ W\text{-}K}$	1	0.968 [0.708, 1.322]	N/A
Pearl-DGS vs SRK/T	1	1.015 [0.636, 1.620]	N/A
Pearl-DGS vs $SRK/T_{modified\ W\text{-}K}$	1	1.000 [0.627, 1.595]	N/A
Pearl-DGS vs $SRK/T_{original\ W\text{-}K}$	0	N/A	N/A
SRK/T vs $SRK/T_{modified\ W\text{-}K}$	4	0.969 [0.803, 1.169]	0.00
SRK/T vs $SRK/T_{original\ W\text{-}K}$	2	0.959 [0.761, 1.208]	0.00
$SRK/T_{modified\ W\text{-}K}$ vs $SRK/T_{original\ W\text{-}K}$	3	0.998 [0.856, 1.164]	0.00

OR: odds ratio; CI: confidence interval; I^2: I-squared heterogeneity statistic; N/A: not applicable
