# Supplementary material for: Evaluation of Intraocular Lens Power Calculation Formulas for Highly Myopic Eyes: A Systematic Review and Network Meta-Analysis
Source: J Clin Med. 2026 Jul 24;15(15):5792. doi: 10.3390/jcm15155792 (PMC13467532; doi:10.3390/jcm15155792)
Supplement: Supplementary file 1 [file jcm-15-05792-s001.zip › Additional File S4.pdf]

Additional File S4. Results of the pairwise network meta-analysis comparing formulas for the percentage of eyes with refractive prediction errors ≤ 0.25 D

**Formula comparison**	**OR [CI 95%]**
EVO vs Barrett Universal II	1.03 [0.88, 1.22]
Haigis vs Barrett Universal II	0.90 [0.76, 1.06]
Hill RBF 2.0 vs Barrett Universal II	1.06 [0.87, 1.28]
Hill RBF 3.0 vs Barrett Universal II	1.30 [0.99, 1.70]
Hoffer Q vs Barrett Universal II	0.64 [0.46, 0.89]
Hoffer QST vs Barrett Universal II	0.86 [0.65, 1.14]
Holladay 1 vs Barrett Universal II	0.70 [0.57, 0.86]
Holladay $1_{\text{modified W-K}}$ vs Barrett Universal II	1.01 [0.85, 1.21]
Holladay $1_{\text{original W-K}}$ vs Barrett Universal II	1.14 [0.94, 1.38]
Kane vs Barrett Universal II	1.18 [1.00, 1.40]
LSF vs Barrett Universal II	0.69 [0.50, 0.94]
Pearl-DGS vs Barrett Universal II	0.95 [0.72, 1.24]
SRK/T vs Barrett Universal II	0.85 [0.71, 1.01]
SRK/$T_{\text{modified W-K}}$ vs Barrett Universal II	0.79 [0.65, 0.94]
SRK/$T_{\text{original W-K}}$ vs Barrett Universal II	0.88 [0.72, 1.07]
Haigis vs EVO	0.87 [0.73, 1.05]
Hill RBF 2.0 vs EVO	1.02 [0.83, 1.25]
Hill RBF 3.0 vs EVO	1.26 [0.96, 1.65]
Hoffer Q vs EVO	0.62 [0.44, 0.87]
Hoffer QST vs EVO	0.83 [0.63, 1.11]
Holladay 1 vs EVO	0.68 [0.54, 0.84]
Holladay $1_{\text{modified W-K}}$ vs EVO	0.98 [0.81, 1.19]
Holladay $1_{\text{original W-K}}$ vs EVO	1.10 [0.90, 1.35]
Kane vs EVO	1.14 [0.96, 1.37]
LSF vs EVO	0.67 [0.48, 0.92]
Pearl-DGS vs EVO	0.92 [0.70, 1.21]
SRK/T vs EVO	0.82 [0.68, 0.99]
SRK/$T_{\text{modified W-K}}$ vs EVO	0.76 [0.63, 0.92]
SRK/$T_{\text{original W-K}}$ vs EVO	0.85 [0.69, 1.05]
Hill RBF 2.0 vs Haigis	1.17 [0.96, 1.43]
Hill RBF 3.0 vs Haigis	1.44 [1.08, 1.92]
Hoffer Q vs Haigis	0.71 [0.51, 0.98]
Hoffer QST vs Haigis	0.95 [0.71, 1.29]
Holladay 1 vs Haigis	0.77 [0.63, 0.96]
Holladay $1_{\text{modified W-K}}$ vs Haigis	1.12 [0.93, 1.36]
Holladay $1_{\text{original W-K}}$ vs Haigis	1.26 [1.03, 1.55]
Kane vs Haigis	1.31 [1.09, 1.57]
LSF vs Haigis	0.76 [0.55, 1.05]
Pearl-DGS vs Haigis	1.05 [0.79, 1.40]
SRK/T vs Haigis	0.94 [0.78, 1.13]
SRK/$T_{\text{modified W-K}}$ vs Haigis	0.87 [0.72, 1.06]
SRK/$T_{\text{original W-K}}$ vs Haigis	0.98 [0.80, 1.20]
Hill RBF 3.0 vs Hill RBF 2.0	1.23 [0.91, 1.66]
Hoffer Q vs Hill RBF 2.0	0.60 [0.43, 0.85]
Hoffer QST vs Hill RBF 2.0	0.82 [0.59, 1.12]
Holladay 1 vs Hill RBF 2.0	0.66 [0.52, 0.83]
Holladay $1_{\text{modified W-K}}$ vs Hill RBF 2.0	0.96 [0.78, 1.18]
Holladay $1_{\text{original W-K}}$ vs Hill RBF 2.0	1.08 [0.87, 1.34]
Kane vs Hill RBF 2.0	1.12 [0.91, 1.38]
LSF vs Hill RBF 2.0	0.65 [0.47, 0.91]
Pearl-DGS vs Hill RBF 2.0	0.90 [0.66, 1.22]

SRK/T vs Hill RBF 2.0	0.80 [0.65, 0.99]
SRK/T$_{\text{modified W-K}}$ vs Hill RBF 2.0	0.74 [0.60, 0.92]
SRK/T$_{\text{original W-K}}$ vs Hill RBF 2.0	0.83 [0.67, 1.04]
Hoffer Q vs Hill RBF 3.0	0.49 [0.33, 0.74]
Hoffer QST vs Hill RBF 3.0	0.66 [0.48, 0.92]
Holladay 1 vs Hill RBF 3.0	0.54 [0.39, 0.73]
Holladay 1$_{\text{modified W-K}}$ vs Hill RBF 3.0	0.78 [0.58, 1.05]
Holladay 1$_{\text{original W-K}}$ vs Hill RBF 3.0	0.88 [0.65, 1.19]
Kane vs Hill RBF 3.0	0.91 [0.69, 1.20]
LSF vs Hill RBF 3.0	0.53 [0.36, 0.78]
Pearl-DGS vs Hill RBF 3.0	0.73 [0.53, 1.00]
SRK/T vs Hill RBF 3.0	0.65 [0.49, 0.87]
SRK/T$_{\text{modified W-K}}$ vs Hill RBF 3.0	0.60 [0.45, 0.81]
SRK/T$_{\text{original W-K}}$ vs Hill RBF 3.0	0.68 [0.50, 0.92]
Hoffer QST vs Hoffer Q	1.35 [0.89, 2.06]
Holladay 1 vs Hoffer Q	1.10 [0.78, 1.54]
Holladay 1$_{\text{modified W-K}}$ vs Hoffer Q	1.59 [1.12, 2.26]
Holladay 1$_{\text{original W-K}}$ vs Hoffer Q	1.79 [1.25, 2.56]
Kane vs Hoffer Q	1.85 [1.31, 2.63]
LSF vs Hoffer Q	1.08 [0.70, 1.67]
Pearl-DGS vs Hoffer Q	1.49 [0.99, 2.25]
SRK/T vs Hoffer Q	1.33 [0.95, 1.85]
SRK/T$_{\text{modified W-K}}$ vs Hoffer Q	1.23 [0.87, 1.75]
SRK/T$_{\text{original W-K}}$ vs Hoffer Q	1.38 [0.96, 1.98]
Holladay 1 vs Hoffer QST	0.81 [0.59, 1.12]
Holladay 1$_{\text{modified W-K}}$ vs Hoffer QST	1.18 [0.86, 1.61]
Holladay 1$_{\text{original W-K}}$ vs Hoffer QST	1.32 [0.96, 1.82]
Kane vs Hoffer QST	1.37 [1.03, 1.83]
LSF vs Hoffer QST	0.80 [0.53, 1.20]
Pearl-DGS vs Hoffer QST	1.10 [0.79, 1.54]
SRK/T vs Hoffer QST	0.98 [0.72, 1.34]
SRK/T$_{\text{modified W-K}}$ vs Hoffer QST	0.91 [0.67, 1.25]
SRK/T$_{\text{original W-K}}$ vs Hoffer QST	1.02 [0.74, 1.41]
Holladay 1$_{\text{modified W-K}}$ vs Holladay 1	1.45 [1.16, 1.82]
Holladay 1$_{\text{original W-K}}$ vs Holladay 1	1.63 [1.29, 2.07]
Kane vs Holladay 1	1.69 [1.35, 2.12]
LSF vs Holladay 1	0.98 [0.70, 1.38]
Pearl-DGS vs Holladay 1	1.36 [1.00, 1.86]
SRK/T vs Holladay 1	1.21 [0.98, 1.50]
SRK/T$_{\text{modified W-K}}$ vs Holladay 1	1.12 [0.90, 1.41]
SRK/T$_{\text{original W-K}}$ vs Holladay 1	1.26 [0.99, 1.60]
Holladay 1$_{\text{original W-K}}$ vs Holladay 1$_{\text{modified W-K}}$	1.13 [0.91, 1.39]
Kane vs Holladay 1$_{\text{modified W-K}}$	1.16 [0.96, 1.42]
LSF vs Holladay 1$_{\text{modified W-K}}$	0.68 [0.49, 0.94]
Pearl-DGS vs Holladay 1$_{\text{modified W-K}}$	0.94 [0.70, 1.26]
SRK/T vs Holladay 1$_{\text{modified W-K}}$	0.83 [0.68, 1.02]
SRK/T$_{\text{modified W-K}}$ vs Holladay 1$_{\text{modified W-K}}$	0.77 [0.63, 0.95]
SRK/T$_{\text{original W-K}}$ vs Holladay 1$_{\text{modified W-K}}$	0.87 [0.70, 1.07]
Kane vs Holladay 1$_{\text{original W-K}}$	1.03 [0.84, 1.28]
LSF vs Holladay 1$_{\text{original W-K}}$	0.60 [0.43, 0.84]
Pearl-DGS vs Holladay 1$_{\text{original W-K}}$	0.83 [0.61, 1.13]
SRK/T vs Holladay 1$_{\text{original W-K}}$	0.74 [0.60, 0.92]
SRK/T$_{\text{modified W-K}}$ vs Holladay 1$_{\text{original W-K}}$	0.69 [0.56, 0.85]
SRK/T$_{\text{original W-K}}$ vs Holladay 1$_{\text{original W-K}}$	0.77 [0.62, 0.96]
LSF vs Kane	0.58 [0.42, 0.81]

Pearl-DGS vs Kane	0.80 [0.61, 1.06]
SRK/T vs Kane	0.72 [0.59, 0.87]
SRK/$T_{modified\ W\text{-}K}$ vs Kane	0.67 [0.54, 0.81]
SRK/$T_{original\ W\text{-}K}$ vs Kane	0.75 [0.60, 0.92]
Pearl-DGS vs LSF	1.38 [0.93, 2.05]
SRK/T vs LSF	1.23 [0.89, 1.70]
SRK/$T_{modified\ W\text{-}K}$ vs LSF	1.14 [0.83, 1.58]
SRK/$T_{original\ W\text{-}K}$ vs LSF	1.28 [0.92, 1.78]
SRK/T vs Pearl-DGS	0.89 [0.66, 1.20]
SRK/$T_{modified\ W\text{-}K}$ vs Pearl-DGS	0.83 [0.61, 1.11]
SRK/$T_{original\ W\text{-}K}$ vs Pearl-DGS	0.93 [0.68, 1.26]
SRK/$T_{modified\ W\text{-}K}$ vs SRK/T	0.93 [0.76, 1.14]
SRK/$T_{original\ W\text{-}K}$ vs SRK/T	1.04 [0.84, 1.30]
SRK/$T_{original\ W\text{-}K}$ vs SRK/$T_{modified\ W\text{-}K}$	1.12 [0.91, 1.39]

OR, odds ratio; CI, confidence interval
