# Supplementary material for: Evaluation of Intraocular Lens Power Calculation Formulas for Highly Myopic Eyes: A Systematic Review and Network Meta-Analysis
Source: J Clin Med. 2026 Jul 24;15(15):5792. doi: 10.3390/jcm15155792 (PMC13467532; doi:10.3390/jcm15155792)
Supplement: Supplementary file 1 [file jcm-15-05792-s001.zip › Additional File S5.pdf]

Additional File S5. Results of the pairwise network meta-analysis comparing formulas for the percentage of eyes with refractive prediction errors ≤ 0.50 D

**Formula comparison**	**OR (CI 95%)**
EVO vs Barrett Universal II	1.11 [0.92, 1.33]
Haigis vs Barrett Universal II	0.91 [0.76, 1.09]
Hill RBF 2.0 vs Barrett Universal II	1.06 [0.86, 1.31]
Hill RBF 3.0 vs Barrett Universal II	1.04 [0.76, 1.42]
Hoffer Q vs Barrett Universal II	0.54 [0.38, 0.76]
Hoffer QST vs Barrett Universal II	0.92 [0.68, 1.25]
Holladay 1 vs Barrett Universal II	0.63 [0.51, 0.78]
Holladay $1_{modified\ W\text{-}K}$ vs Barrett Universal II	1.03 [0.84, 1.26]
Holladay $1_{original\ W\text{-}K}$ vs Barrett Universal II	1.20 [0.96, 1.49]
Kane vs Barrett Universal II	1.19 [0.98, 1.44]
LSF vs Barrett Universal II	0.62 [0.46, 0.84]
Pearl-DGS vs Barrett Universal II	0.91 [0.67, 1.23]
SRK/T vs Barrett Universal II	0.76 [0.63, 0.92]
SRK/$T_{modified\ W\text{-}K}$ vs Barrett Universal II	0.91 [0.75, 1.11]
SRK/$T_{original\ W\text{-}K}$ vs Barrett Universal II	0.95 [0.77, 1.17]
Haigis vs EVO	0.82 [0.67, 1.00]
Hill RBF 2.0 vs EVO	0.96 [0.76, 1.20]
Hill RBF 3.0 vs EVO	0.94 [0.68, 1.29]
Hoffer Q vs EVO	0.49 [0.34, 0.70]
Hoffer QST vs EVO	0.83 [0.61, 1.14]
Holladay 1 vs EVO	0.57 [0.45, 0.71]
Holladay $1_{modified\ W\text{-}K}$ vs EVO	0.93 [0.75, 1.16]
Holladay $1_{original\ W\text{-}K}$ vs EVO	1.08 [0.86, 1.37]
Kane vs EVO	1.07 [0.87, 1.33]
LSF vs EVO	0.56 [0.41, 0.77]
Pearl-DGS vs EVO	0.82 [0.60, 1.12]
SRK/T vs EVO	0.69 [0.56, 0.85]
SRK/$T_{modified\ W\text{-}K}$ vs EVO	0.83 [0.67, 1.02]
SRK/$T_{original\ W\text{-}K}$ vs EVO	0.86 [0.68, 1.08]
Hill RBF 2.0 vs Haigis	1.17 [0.94, 1.45]
Hill RBF 3.0 vs Haigis	1.15 [0.83, 1.59]
Hoffer Q vs Haigis	0.60 [0.42, 0.84]
Hoffer QST vs Haigis	1.02 [0.74, 1.40]
Holladay 1 vs Haigis	0.69 [0.56, 0.86]
Holladay $1_{modified\ W\text{-}K}$ vs Haigis	1.14 [0.92, 1.40]
Holladay $1_{original\ W\text{-}K}$ vs Haigis	1.32 [1.05, 1.65]
Kane vs Haigis	1.31 [1.07, 1.61]
LSF vs Haigis	0.68 [0.50, 0.93]
Pearl-DGS vs Haigis	1.00 [0.72, 1.38]
SRK/T vs Haigis	0.84 [0.69, 1.01]
SRK/$T_{modified\ W\text{-}K}$ vs Haigis	1.01 [0.82, 1.23]
SRK/$T_{original\ W\text{-}K}$ vs Haigis	1.05 [0.84, 1.30]
Hill RBF 3.0 vs Hill RBF 2.0	0.98 [0.69, 1.39]
Hoffer Q vs Hill RBF 2.0	0.51 [0.35, 0.74]
Hoffer QST vs Hill RBF 2.0	0.87 [0.62, 1.23]
Holladay 1 vs Hill RBF 2.0	0.59 [0.46, 0.76]
Holladay $1_{modified\ W\text{-}K}$ vs Hill RBF 2.0	0.97 [0.77, 1.23]
Holladay $1_{original\ W\text{-}K}$ vs Hill RBF 2.0	1.13 [0.88, 1.45]
Kane vs Hill RBF 2.0	1.12 [0.89, 1.42]
LSF vs Hill RBF 2.0	0.59 [0.42, 0.81]
Pearl-DGS vs Hill RBF 2.0	0.86 [0.61, 1.21]

SRK/T vs Hill RBF 2.0	0.72 [0.57, 0.90]
SRK/T$_{\text{modified W-K}}$ vs Hill RBF 2.0	0.86 [0.69, 1.08]
SRK/T$_{\text{original W-K}}$ vs Hill RBF 2.0	0.90 [0.71, 1.14]
Hoffer Q vs Hill RBF 3.0	0.52 [0.33, 0.81]
Hoffer QST vs Hill RBF 3.0	0.89 [0.61, 1.28]
Holladay 1 vs Hill RBF 3.0	0.60 [0.43, 0.85]
Holladay 1$_{\text{modified W-K}}$ vs Hill RBF 3.0	0.99 [0.71, 1.39]
Holladay 1$_{\text{original W-K}}$ vs Hill RBF 3.0	1.15 [0.81, 1.63]
Kane vs Hill RBF 3.0	1.14 [0.83, 1.57]
LSF vs Hill RBF 3.0	0.60 [0.39, 0.90]
Pearl-DGS vs Hill RBF 3.0	0.87 [0.60, 1.25]
SRK/T vs Hill RBF 3.0	0.73 [0.52, 1.02]
SRK/T$_{\text{modified W-K}}$ vs Hill RBF 3.0	0.88 [0.63, 1.23]
SRK/T$_{\text{original W-K}}$ vs Hill RBF 3.0	0.91 [0.64, 1.29]
Hoffer QST vs Hoffer Q	1.71 [1.10, 2.64]
Holladay 1 vs Hoffer Q	1.16 [0.82, 1.64]
Holladay 1$_{\text{modified W-K}}$ vs Hoffer Q	1.91 [1.33, 2.75]
Holladay 1$_{\text{original W-K}}$ vs Hoffer Q	2.21 [1.52, 3.22]
Kane vs Hoffer Q	2.20 [1.52, 3.17]
LSF vs Hoffer Q	1.15 [0.75, 1.76]
Pearl-DGS vs Hoffer Q	1.68 [1.08, 2.60]
SRK/T vs Hoffer Q	1.41 [1.00, 1.98]
SRK/T$_{\text{modified W-K}}$ vs Hoffer Q	1.69 [1.18, 2.43]
SRK/T$_{\text{original W-K}}$ vs Hoffer Q	1.76 [1.21, 2.55]
Holladay 1 vs Hoffer QST	0.68 [0.49, 0.95]
Holladay 1$_{\text{modified W-K}}$ vs Hoffer QST	1.12 [0.80, 1.56]
Holladay 1$_{\text{original W-K}}$ vs Hoffer QST	1.30 [0.92, 1.83]
Kane vs Hoffer QST	1.29 [0.94, 1.77]
LSF vs Hoffer QST	0.67 [0.45, 1.01]
Pearl-DGS vs Hoffer QST	0.98 [0.68, 1.41]
SRK/T vs Hoffer QST	0.82 [0.60, 1.14]
SRK/T$_{\text{modified W-K}}$ vs Hoffer QST	0.99 [0.71, 1.38]
SRK/T$_{\text{original W-K}}$ vs Hoffer QST	1.03 [0.73, 1.45]
Holladay 1$_{\text{modified W-K}}$ vs Holladay 1	1.64 [1.30, 2.07]
Holladay 1$_{\text{original W-K}}$ vs Holladay 1	1.90 [1.48, 2.44]
Kane vs Holladay 1	1.89 [1.49, 2.41]
LSF vs Holladay 1	0.99 [0.71, 1.37]
Pearl-DGS vs Holladay 1	1.44 [1.03, 2.02]
SRK/T vs Holladay 1	1.21 [0.98, 1.50]
SRK/T$_{\text{modified W-K}}$ vs Holladay 1	1.46 [1.16, 1.83]
SRK/T$_{\text{original W-K}}$ vs Holladay 1	1.51 [1.18, 1.93]
Holladay 1$_{\text{original W-K}}$ vs Holladay 1$_{\text{modified W-K}}$	1.16 [0.92, 1.47]
Kane vs Holladay 1$_{\text{modified W-K}}$	1.15 [0.92, 1.44]
LSF vs Holladay 1$_{\text{modified W-K}}$	0.60 [0.44, 0.82]
Pearl-DGS vs Holladay 1$_{\text{modified W-K}}$	0.88 [0.63, 1.23]
SRK/T vs Holladay 1$_{\text{modified W-K}}$	0.74 [0.59, 0.91]
SRK/T$_{\text{modified W-K}}$ vs Holladay 1$_{\text{modified W-K}}$	0.89 [0.72, 1.10]
SRK/T$_{\text{original W-K}}$ vs Holladay 1$_{\text{modified W-K}}$	0.92 [0.73, 1.16]
Kane vs Holladay 1$_{\text{original W-K}}$	0.99 [0.78, 1.27]
LSF vs Holladay 1$_{\text{original W-K}}$	0.52 [0.37, 0.72]
Pearl-DGS vs Holladay 1$_{\text{original W-K}}$	0.76 [0.54, 1.07]
SRK/T vs Holladay 1$_{\text{original W-K}}$	0.64 [0.50, 0.80]
SRK/T$_{\text{modified W-K}}$ vs Holladay 1$_{\text{original W-K}}$	0.77 [0.61, 0.96]
SRK/T$_{\text{original W-K}}$ vs Holladay 1$_{\text{original W-K}}$	0.79 [0.62, 1.01]
LSF vs Kane	0.52 [0.38, 0.72]

Pearl-DGS vs Kane	0.76 [0.56, 1.04]
SRK/T vs Kane	0.64 [0.51, 0.80]
SRK/$T_{modified\ W\text{-}K}$ vs Kane	0.77 [0.62, 0.96]
SRK/$T_{original\ W\text{-}K}$ vs Kane	0.80 [0.63, 1.01]
Pearl-DGS vs LSF	1.46 [0.97, 2.20]
SRK/T vs LSF	1.23 [0.90, 1.68]
SRK/$T_{modified\ W\text{-}K}$ vs LSF	1.48 [1.08, 2.02]
SRK/$T_{original\ W\text{-}K}$ vs LSF	1.53 [1.10, 2.13]
SRK/T vs Pearl-DGS	0.84 [0.60, 1.16]
SRK/$T_{modified\ W\text{-}K}$ vs Pearl-DGS	1.01 [0.73, 1.40]
SRK/$T_{original\ W\text{-}K}$ vs Pearl-DGS	1.05 [0.74, 1.48]
SRK/$T_{modified\ W\text{-}K}$ vs SRK/T	1.20 [0.97, 1.49]
SRK/$T_{original\ W\text{-}K}$ vs SRK/T	1.25 [0.99, 1.57]
SRK/$T_{original\ W\text{-}K}$ vs SRK/$T_{modified\ W\text{-}K}$	1.04 [0.83, 1.30]

OR, odds ratio; CI, confidence interval
