# Supplementary material for: Evaluation of Intraocular Lens Power Calculation Formulas for Highly Myopic Eyes: A Systematic Review and Network Meta-Analysis
Source: J Clin Med. 2026 Jul 24;15(15):5792. doi: 10.3390/jcm15155792 (PMC13467532; doi:10.3390/jcm15155792)
Supplement: Supplementary file 1 [file jcm-15-05792-s001.zip › Additional File S6.pdf]

Additional File S6. Results of the pairwise network meta-analysis comparing formulas for the percentage of eyes with refractive prediction errors <1.00 DP

**Formula comparison**	**OR (CI 95%)**
EVO vs Barrett Universal II	0.87 [0.57, 1.33]
Haigis vs Barrett Universal II	0.67 [0.46, 0.98]
Hill RBF 2.0 vs Barrett Universal II	1.01 [0.63, 1.63]
Hill RBF 3.0 vs Barrett Universal II	0.93 [0.41, 2.11]
Hoffer Q vs Barrett Universal II	0.32 [0.19, 0.53]
Hoffer QST vs Barrett Universal II	0.63 [0.35, 1.13]
Holladay 1 vs Barrett Universal II	0.40 [0.27, 0.60]
Holladay $1_{\text{modified W-K}}$ vs Barrett Universal II	0.80 [0.52, 1.24]
Holladay $1_{\text{original W-K}}$ vs Barrett Universal II	1.24 [0.73, 2.12]
Kane vs Barrett Universal II	1.16 [0.72, 1.86]
LSF vs Barrett Universal II	0.46 [0.28, 0.77]
Pearl-DGS vs Barrett Universal II	1.00 [0.43, 2.35]
SRK/T vs Barrett Universal II	0.51 [0.35, 0.74]
SRK/$T_{\text{modified W-K}}$ vs Barrett Universal II	0.71 [0.47, 1.06]
SRK/$T_{\text{original W-K}}$ vs Barrett Universal II	0.68 [0.44, 1.06]
Haigis vs EVO	0.77 [0.51, 1.16]
Hill RBF 2.0 vs EVO	1.16 [0.71, 1.91]
Hill RBF 3.0 vs EVO	1.07 [0.46, 2.48]
Hoffer Q vs EVO	0.36 [0.21, 0.63]
Hoffer QST vs EVO	0.72 [0.39, 1.33]
Holladay 1 vs EVO	0.46 [0.30, 0.71]
Holladay $1_{\text{modified W-K}}$ vs EVO	0.92 [0.58, 1.46]
Holladay $1_{\text{original W-K}}$ vs EVO	1.43 [0.83, 2.47]
Kane vs EVO	1.33 [0.81, 2.19]
LSF vs EVO	0.53 [0.31, 0.91]
Pearl-DGS vs EVO	1.15 [0.48, 2.76]
SRK/T vs EVO	0.59 [0.39, 0.89]
SRK/$T_{\text{modified W-K}}$ vs EVO	0.81 [0.53, 1.25]
SRK/$T_{\text{original W-K}}$ vs EVO	0.78 [0.49, 1.24]
Hill RBF 2.0 vs Haigis	1.51 [0.96, 2.37]
Hill RBF 3.0 vs Haigis	1.38 [0.60, 3.17]
Hoffer Q vs Haigis	0.47 [0.30, 0.74]
Hoffer QST vs Haigis	0.93 [0.52, 1.69]
Holladay 1 vs Haigis	0.60 [0.42, 0.85]
Holladay $1_{\text{modified W-K}}$ vs Haigis	1.20 [0.80, 1.79]
Holladay $1_{\text{original W-K}}$ vs Haigis	1.85 [1.10, 3.12]
Kane vs Haigis	1.72 [1.08, 2.74]
LSF vs Haigis	0.69 [0.42, 1.12]
Pearl-DGS vs Haigis	1.49 [0.63, 3.51]
SRK/T vs Haigis	0.76 [0.55, 1.05]
SRK/$T_{\text{modified W-K}}$ vs Haigis	1.05 [0.72, 1.54]
SRK/$T_{\text{original W-K}}$ vs Haigis	1.01 [0.66, 1.54]
Hill RBF 3.0 vs Hill RBF 2.0	0.92 [0.38, 2.22]
Hoffer Q vs Hill RBF 2.0	0.31 [0.18, 0.55]
Hoffer QST vs Hill RBF 2.0	0.62 [0.32, 1.22]
Holladay 1 vs Hill RBF 2.0	0.40 [0.25, 0.64]
Holladay $1_{\text{modified W-K}}$ vs Hill RBF 2.0	0.79 [0.48, 1.30]
Holladay $1_{\text{original W-K}}$ vs Hill RBF 2.0	1.23 [0.68, 2.21]
Kane vs Hill RBF 2.0	1.14 [0.67, 1.96]
LSF vs Hill RBF 2.0	0.46 [0.26, 0.81]
Pearl-DGS vs Hill RBF 2.0	0.99 [0.40, 2.46]

SRK/T vs Hill RBF 2.0	0.50 [0.32, 0.80]
SRK/$T_{modified\ W\text{-}K}$ vs Hill RBF 2.0	0.70 [0.43, 1.12]
SRK/$T_{original\ W\text{-}K}$ vs Hill RBF 2.0	0.67 [0.41, 1.10]
Hoffer Q vs Hill RBF 3.0	0.34 [0.14, 0.84]
Hoffer QST vs Hill RBF 3.0	0.67 [0.29, 1.59]
Holladay 1 vs Hill RBF 3.0	0.43 [0.19, 1.00]
Holladay $1_{modified\ W\text{-}K}$ vs Hill RBF 3.0	0.86 [0.37, 2.03]
Holladay $1_{original\ W\text{-}K}$ vs Hill RBF 3.0	1.34 [0.53, 3.35]
Kane vs Hill RBF 3.0	1.24 [0.53, 2.91]
LSF vs Hill RBF 3.0	0.50 [0.20, 1.23]
Pearl-DGS vs Hill RBF 3.0	1.08 [0.38, 3.03]
SRK/T vs Hill RBF 3.0	0.55 [0.24, 1.26]
SRK/$T_{modified\ W\text{-}K}$ vs Hill RBF 3.0	0.76 [0.33, 1.77]
SRK/$T_{original\ W\text{-}K}$ vs Hill RBF 3.0	0.73 [0.31, 1.74]
Hoffer QST vs Hoffer Q	1.98 [0.99, 3.96]
Holladay 1 vs Hoffer Q	1.27 [0.82, 1.98]
Holladay $1_{modified\ W\text{-}K}$ vs Hoffer Q	2.54 [1.48, 4.38]
Holladay $1_{original\ W\text{-}K}$ vs Hoffer Q	3.93 [2.09, 7.42]
Kane vs Hoffer Q	3.66 [2.03, 6.61]
LSF vs Hoffer Q	1.46 [0.80, 2.67]
Pearl-DGS vs Hoffer Q	3.17 [1.25, 8.02]
SRK/T vs Hoffer Q	1.62 [1.03, 2.53]
SRK/$T_{modified\ W\text{-}K}$ vs Hoffer Q	2.24 [1.32, 3.79]
SRK/$T_{original\ W\text{-}K}$ vs Hoffer Q	2.15 [1.24, 3.74]
Holladay 1 vs Hoffer QST	0.64 [0.35, 1.17]
Holladay $1_{modified\ W\text{-}K}$ vs Hoffer QST	1.28 [0.67, 2.43]
Holladay $1_{original\ W\text{-}K}$ vs Hoffer QST	1.98 [0.97, 4.07]
Kane vs Hoffer QST	1.85 [0.97, 3.52]
LSF vs Hoffer QST	0.74 [0.37, 1.48]
Pearl-DGS vs Hoffer QST	1.60 [0.65, 3.95]
SRK/T vs Hoffer QST	0.81 [0.45, 1.47]
SRK/$T_{modified\ W\text{-}K}$ vs Hoffer QST	1.13 [0.60, 2.11]
SRK/$T_{original\ W\text{-}K}$ vs Hoffer QST	1.08 [0.57, 2.08]
Holladay $1_{modified\ W\text{-}K}$ vs Holladay 1	1.99 [1.29, 3.08]
Holladay $1_{original\ W\text{-}K}$ vs Holladay 1	3.09 [1.80, 5.30]
Kane vs Holladay 1	2.87 [1.76, 4.71]
LSF vs Holladay 1	1.15 [0.69, 1.90]
Pearl-DGS vs Holladay 1	2.49 [1.05, 5.91]
SRK/T vs Holladay 1	1.27 [0.91, 1.77]
SRK/$T_{modified\ W\text{-}K}$ vs Holladay 1	1.75 [1.16, 2.65]
SRK/$T_{original\ W\text{-}K}$ vs Holladay 1	1.69 [1.08, 2.63]
Holladay $1_{original\ W\text{-}K}$ vs Holladay $1_{modified\ W\text{-}K}$	1.55 [0.89, 2.69]
Kane vs Holladay $1_{modified\ W\text{-}K}$	1.44 [0.87, 2.38]
LSF vs Holladay $1_{modified\ W\text{-}K}$	0.58 [0.35, 0.96]
Pearl-DGS vs Holladay $1_{modified\ W\text{-}K}$	1.25 [0.52, 3.01]
SRK/T vs Holladay $1_{modified\ W\text{-}K}$	0.64 [0.42, 0.96]
SRK/$T_{modified\ W\text{-}K}$ vs Holladay $1_{modified\ W\text{-}K}$	0.88 [0.57, 1.35]
SRK/$T_{original\ W\text{-}K}$ vs Holladay $1_{modified\ W\text{-}K}$	0.85 [0.53, 1.34]
Kane vs Holladay $1_{original\ W\text{-}K}$	0.93 [0.51, 1.69]
LSF vs Holladay $1_{original\ W\text{-}K}$	0.37 [0.20, 0.69]
Pearl-DGS vs Holladay $1_{original\ W\text{-}K}$	0.81 [0.31, 2.07]
SRK/T vs Holladay $1_{original\ W\text{-}K}$	0.41 [0.24, 0.69]
SRK/$T_{modified\ W\text{-}K}$ vs Holladay $1_{original\ W\text{-}K}$	0.57 [0.33, 0.97]
SRK/$T_{original\ W\text{-}K}$ vs Holladay $1_{original\ W\text{-}K}$	0.55 [0.32, 0.95]
LSF vs Kane	0.40 [0.22, 0.71]

Pearl-DGS vs Kane	0.87 [0.36, 2.09]
SRK/T vs Kane	0.44 [0.28, 0.71]
SRK/$T_{modified\ W\text{-}K}$ vs Kane	0.61 [0.38, 0.99]
SRK/$T_{original\ W\text{-}K}$ vs Kane	0.59 [0.35, 0.98]
Pearl-DGS vs LSF	2.17 [0.86, 5.48]
SRK/T vs LSF	1.11 [0.68, 1.79]
SRK/$T_{modified\ W\text{-}K}$ vs LSF	1.53 [0.92, 2.53]
SRK/$T_{original\ W\text{-}K}$ vs LSF	1.47 [0.86, 2.52]
SRK/T vs Pearl-DGS	0.51 [0.22, 1.20]
SRK/$T_{modified\ W\text{-}K}$ vs Pearl-DGS	0.71 [0.29, 1.69]
SRK/$T_{original\ W\text{-}K}$ vs Pearl-DGS	0.68 [0.28, 1.66]
SRK/$T_{modified\ W\text{-}K}$ vs SRK/T	1.38 [0.94, 2.03]
SRK/$T_{original\ W\text{-}K}$ vs SRK/T	1.33 [0.87, 2.03]
SRK/$T_{original\ W\text{-}K}$ vs SRK/$T_{modified\ W\text{-}K}$	0.96 [0.62, 1.48]

OR, odds ratio; CI, confidence interval
