# Supplementary material for: Evaluation of Intraocular Lens Power Calculation Formulas for Highly Myopic Eyes: A Systematic Review and Network Meta-Analysis
Source: J Clin Med. 2026 Jul 24;15(15):5792. doi: 10.3390/jcm15155792 (PMC13467532; doi:10.3390/jcm15155792)
Supplement: Supplementary file 1 [file jcm-15-05792-s001.zip › Additional File S7.pdf]

Additional File S7. Node-split analysis of inconsistency for the percentage of eyes with a refractive prediction error ≤ 0.25 D

Comparison	Direct Estimate	Direct SE	Indirect Estimate	Indirect SE	Difference Estimate	Difference Standard Error	P>z
Barrett Universal II vs EVO	0.0758595	0.0880462	−0.3615171	0.2644574	0.4373767	0.2788318	0.117
Barrett Universal II vs Haigis	−0.1446072	0.0909311	0.1469778	0.224647	−0.2915849	0.2422579	0.229
Barrett Universal II vs Hill RBF 2.0	−0.0227832	0.1075587	0.37179	0.2183293	−0.3945732	0.2431215	0.105
Barrett Universal II vs Hill RBF 3.0	0.3791628	0.1626885	−0.032574	0.2575873	0.4117367	0.3059293	0.178
Barrett Universal II vs Hoffer Q	−0.7008559	0.2552692	−0.2531007	0.2274808	−0.4477553	0.340476	0.188
Barrett Universal II vs Hoffer QST	0.0876643	0.1741139	−0.7151029	0.2705458	0.8027672	0.3244302	0.013
Barrett Universal II vs Holladay 1	−0.3964399	0.1247188	−0.2729402	0.1893212	−0.1234996	0.2257318	0.584
Barrett Universal II vs Holladay $1_{\text{modified W-K}}$	−0.0395731	0.1001674	0.290477	0.2294657	−0.3300501	0.2501138	0.187
Barrett Universal II vs Holladay $1_{\text{original W-K}}$	0.0822128	0.1101729	0.3208434	0.2170596	−0.2386306	0.2436083	0.327
Barrett Universal II vs Kane	0.1865971	0.0934215	0.031556	0.2370547	0.1550411	0.2551478	0.543
Barrett Universal II vs LSF	−0.3992432	0.1936699	−0.3307368	0.2576949	−0.0685064	0.311648	0.826
Barrett Universal II vs Pearl-DGS	0.0659867	0.1634078	−0.34575	0.2579353	0.4117367	0.3059293	0.178
Barrett Universal II vs SRK/T	−0.1955737	0.1020747	−0.0629409	0.1988205	−0.1326327	0.2233227	0.553
Barrett Universal II vs $\text{SRK/T}_{\text{modified W-K}}$	−0.2946109	0.1014367	0.0354392	0.2299827	−0.3300501	0.2501137	0.187

Barrett Universal II vs SRK/T$_{original\ W\text{-}K}$	−0.17604	0.1114203	0.0625906	0.2176993	−0.2386306	0.2436083	0.327
EVO vs Haigis	−0.0082629	0.1114585	−0.4032133	0.1614674	0.3949504	0.1962109	0.044
EVO vs Hill RBF 2.0	0.0659696	0.1496129	−0.0196107	0.1452952	0.0855803	0.2086413	0.682
EVO vs Hill RBF 3.0	0.1037454	0.1612716	0.5790095	0.2696143	−0.475264	0.3141013	0.130
EVO vs Hoffer QST	−0.2712371	0.1713468	0.0554671	0.2796317	−0.3267042	0.3274358	0.318
EVO vs Holladay 1	−0.4019941	0.1735282	−0.3839224	0.1453245	−0.0180717	0.2257243	0.936
EVO vs Holladay 1$_{modified\ W\text{-}K}$	0.0416869	0.1164699	−0.1684199	0.1826128	0.2101067	0.216599	0.332
EVO vs Holladay 1$_{original\ W\text{-}K}$	0.1819757	0.1235094	−0.10189	0.1926322	0.2838657	0.229134	0.215
EVO vs Kane	0.1274027	0.0974153	0.1778551	0.2628438	−0.0504524	0.2804358	0.857
EVO vs LSF	−0.3351308	0.2247068	−0.4859826	0.2345776	0.1508518	0.3225172	0.640
EVO vs Pearl-DGS	−0.2096815	0.1619995	0.2655825	0.2699508	−0.475264	0.3141013	0.130
EVO vs SRK/T	−0.188614	0.1340094	−0.2133493	0.144475	0.0247353	0.1970239	0.900
EVO vs SRK/T$_{modified\ W\text{-}K}$	−0.2535309	0.1180236	−0.3241188	0.1840532	0.070588	0.2183493	0.746
EVO vs SRK/T$_{original\ W\text{-}K}$	−0.0773589	0.1247207	−0.3591676	0.1948991	0.2818087	0.231203	0.223
Haigis vs Hill RBF 2.0	0.1835751	0.1085598	0.002216	0.2671847	0.1813591	0.2888893	0.530
Haigis vs Hill RBF 3.0	0.0552686	0.3336554	0.4375229	0.1618826	−0.3822543	0.3708878	0.303
Haigis vs Hoffer Q	−0.2785986	0.199441	−0.5151687	0.3085883	0.2365701	0.364181	0.516
Haigis vs Hoffer QST	−0.271972	0.4164873	−0.0122301	0.1643631	−0.2597419	0.4461714	0.560
Haigis vs Holladay 1	−0.2466012	0.1344528	−0.2713677	0.1770621	0.0247665	0.2215288	0.911
Haigis vs Holladay 1$_{modified\ W\text{-}K}$	0.1798489	0.1129478	−0.0692915	0.1940754	0.2491404	0.2248349	0.268
Haigis vs Holladay 1$_{original\ W\text{-}K}$	0.1900238	0.1275338	0.3217331	0.1777395	−0.1317093	0.2190662	0.548
Haigis vs Kane	0.3036577	0.1088834	0.164274	0.1905493	0.1393837	0.2198005	0.526
Haigis vs LSF	−0.3720942	0.399236	−0.2530629	0.1777531	−0.1190312	0.4305055	0.782
Haigis vs Pearl-DGS	0.1113373	0.333591	0.0370603	0.1628913	0.074277	0.3712839	0.841

Haigis vs SRK/T	−0.0431181	0.1079993	−0.1332036	0.194671	0.0900855	0.2226473	0.686
Haigis vs SRK/$T_{modified\ W\text{-}K}$	−0.0931958	0.1143083	−0.2712462	0.1960601	0.1780504	0.2268613	0.433
Haigis vs SRK/$T_{original\ W\text{-}K}$	-0.1227407	0.1293434	0.1655149	0.1781783	-0.2882556	0.2198137	0.190
Hill RBF 2.0 vs Hoffer Q	−0.8637507	0.2568918	−0.1801571	0.2445434	−0.6835935	0.3542167	0.054
Hill RBF 2.0 vs Holladay 1	−0.427039	0.1793333	−0.4026544	0.1577563	−0.0243846	0.2387724	0.919
Hill RBF 2.0 vs Holladay $1_{modified\ W\text{-}K}$	0.0472304	0.118834	−0.3889646	0.2359458	0.436195	0.2641505	0.099
Hill RBF 2.0 vs Holladay $1_{original\ W\text{-}K}$	0.158872	0.1273777	−0.1863707	0.22885	0.3452427	0.2619434	0.188
Hill RBF 2.0 vs Kane	0.2050772	0.1345393	−0.0412373	0.1719595	0.2463144	0.2182118	0.259
Hill RBF 2.0 vs LSF	−1.118093	0.3711556	−0.27236	0.1861609	−0.8457326	0.4048252	0.037
Hill RBF 2.0 vs SRK/T	−0.2975389	0.1570019	−0.1546383	0.1482179	−0.1429006	0.2159489	0.508
Hill RBF 2.0 vs SRK/$T_{modified\ W\text{-}K}$	−0.2086364	0.1204772	−0.6356142	0.2381186	0.4269778	0.2672064	0.110
Hill RBF 2.0 vs SRK/$T_{original\ W\text{-}K}$	−0.1508799	0.1291874	−0.276227	0.2293042	0.1253471	0.2631326	0.634
Hill RBF 3.0 vs Hoffer QST	−0.4982604	0.1860388	−0.0016293	0.4029528	−0.496631	0.4435169	0.263
Hill RBF 3.0 vs Holladay 1	−0.4405638	0.3387931	−0.6707852	0.1790549	0.2302215	0.3838524	0.549
Hill RBF 3.0 vs Holladay $1_{modified\ W\text{-}K}$	0.2794897	0.3344338	−0.3811391	0.1678839	0.6606288	0.3743629	0.078
Hill RBF 3.0 vs Kane	−0.1560106	0.1615408	0.0845342	0.2786415	−0.2405448	0.3222892	0.455
Hill RBF 3.0 vs Pearl-DGS	N/A	N/A	N/A	N/A	N/A	N/A	N/A
Hill RBF 3.0 vs SRK/T	−0.1104817	0.3339787	−0.5089496	0.167269	0.3984679	0.3736088	0.286
Hill RBF 3.0 vs SRK/$T_{modified\ W\text{-}K}$	−0.110694	0.3339787	−0.6035045	0.1689826	0.4928105	0.3743761	0.188
Hoffer Q vs Holladay 1	0.0161197	0.2035881	0.3074497	0.3443609	−0.29133	0.400673	0.467
Hoffer Q vs SRK/T	0.2000771	0.2015282	0.4902932	0.3171096	−0.2902161	0.3743235	0.438

Hoffer QST vs Holladay 1	0.1160716	0.4241602	−0.2666034	0.1789781	0.382675	0.4598445	0.405
Hoffer QST vs Kane	0.2246764	0.1862509	0.4707512	0.2424222	−0.2460748	0.3046901	0.419
Hoffer QST vs Pearl-DGS	0.0701965	0.1871339	0.2266028	0.4028899	−0.1564063	0.4441212	0.725
Hoffer QST vs SRK/T	0.3853087	0.4122206	−0.0837588	0.1689309	0.4690675	0.4435667	0.290
Holladay 1 vs Holladay $1_{modified\ W\text{-}K}$	0.2740622	0.1524515	0.5023889	0.1753446	−0.2283266	0.2318912	0.325
Holladay 1 vs Holladay $1_{original\ W\text{-}K}$	0.4053197	0.1687831	0.577508	0.1706149	−0.1721884	0.2396367	0.472
Holladay 1 vs Kane	0.6740898	0.3393151	0.5052592	0.1233603	0.1688306	0.3607838	0.640
Holladay 1 vs LSF	0.2208175	0.2313593	−0.3193507	0.2623885	0.5401682	0.3521844	0.125
Holladay 1 vs Pearl-DGS	0.5088447	0.3387273	0.2496537	0.1800328	0.259191	0.38428	0.500
Holladay 1 vs SRK/T	0.2054203	0.1166244	0.0805724	0.3229365	0.1248479	0.342303	0.715
Holladay 1 vs $SRK/T_{modified\ W\text{-}K}$	0.0378185	0.1542148	0.2223806	0.1773076	−0.1845621	0.2349777	0.432
Holladay 1 vs $SRK/T_{original\ W\text{-}K}$	0.2425769	0.1702642	0.221597	0.1725263	0.0209799	0.2422016	0.931
Holladay $1_{modified\ W\text{-}K}$ vs Holladay $1_{original\ W\text{-}K}$	0.1491321	0.1106003	−0.2374144	0.3768816	0.3865466	0.3931309	0.325
Holladay $1_{modified\ W\text{-}K}$ vs Kane	0.1171946	0.1247107	0.2208848	0.1732479	−0.1036902	0.2134448	0.627
Holladay $1_{modified\ W\text{-}K}$ vs LSF	−0.2455327	0.1951232	−0.7046787	0.2846145	0.459146	0.3367656	0.173
Holladay $1_{modified\ W\text{-}K}$ vs Pearl-DGS	−0.2214717	0.3343691	−0.0253848	0.1688802	−0.1960868	0.3747673	0.601
Holladay $1_{modified\ W\text{-}K}$ vs SRK/T	−0.1045651	0.1374233	−0.2836818	0.1593092	0.1791167	0.2103866	0.395
Holladay $1_{modified\ W\text{-}K}$ vs $SRK/T_{modified\ W\text{-}K}$	N/A	N/A	N/A	N/A	N/A	N/A	N/A
Holladay $1_{modified\ W\text{-}K}$	−0.1091223	0.1118448	−0.4956689	0.3772469	0.3865466	0.3931309	0.325

vs SRK/T$_{\text{original W-K}}$							
Holladay 1$_{\text{original W-K}}$ vs Kane	0.0652868	0.1473232	−0.0007757	0.157189	0.0660625	0.2154144	0.759
Holladay 1$_{\text{original W-K}}$ vs LSF	−0.3589223	0.2245991	−0.6899497	0.2502224	0.3310274	0.3340265	0.322
Holladay 1$_{\text{original W-K}}$ vs SRK/T	−0.230931	0.1666735	−0.3520218	0.1466579	0.1210908	0.2221386	0.586
Holladay 1$_{\text{original W-K}}$ vs SRK/T$_{\text{modified W-K}}$	−0.4214466	0.1123569	0.1501485	0.3769607	−0.5715951	0.394698	0.148
Holladay 1$_{\text{original W-K}}$ vs SRK/T$_{\text{original W-K}}$	N/A	N/A	N/A	N/A	N/A	N/A	N/A
Kane vs LSF	−1.06781	0.3713758	−0.4292758	0.1810756	−0.6385346	0.4019403	0.112
Kane vs Pearl-DGS	−0.1572251	0.1622675	−0.3977699	0.2789678	0.2405448	0.3222892	0.455
Kane vs SRK/T	−0.3433297	0.1595917	−0.3268503	0.1323625	−0.0164794	0.2072791	0.937
Kane vs SRK/T$_{\text{modified W-K}}$	−0.4047957	0.1260954	−0.4129484	0.1754331	0.0081527	0.2159387	0.970
Kane vs SRK/T$_{\text{original W-K}}$	−0.3988121	0.1492748	−0.1730449	0.1584157	−0.2257672	0.217334	0.299
LSF vs SRK/T	0.1694837	0.1963194	0.290869	0.2874055	−0.1213853	0.3412769	0.722
LSF vs SRK/T$_{\text{modified W-K}}$	0.1253769	0.196917	0.1505313	0.2883407	−0.0251544	0.3429571	0.942
LSF vs SRK/T$_{\text{original W-K}}$	0.2608696	0.225134	0.2320741	0.2524559	0.0287954	0.3364466	0.932
Pearl-DGS vs SRK/T	−0.167331	0.3339142	−0.1025657	0.1682657	−0.0647652	0.3740113	0.863
Pearl-DGS vs SRK/T$_{\text{modified W-K}}$	−0.1675649	0.3339142	−0.1954376	0.1699764	0.0278728	0.3747806	0.941
SRK/T vs SRK/T$_{\text{modified W-K}}$	−0.0889737	0.1387419	−0.0537253	0.1615012	−0.0352484	0.2129149	0.869
SRK/T vs SRK/T$_{\text{original W-K}}$	0.0664221	0.1681388	0.0210399	0.1484194	0.0453821	0.2242855	0.840
SRK/T$_{\text{modified W-K}}$ vs SRK/T$_{\text{original W-K}}$	0.1631925	0.1135818	−0.4084026	0.3773261	0.5715951	0.394698	0.148

SE: standard error; N/A: not applicable; P>z: p value for inconsistency.
