# Supplementary material for: Evaluation of Intraocular Lens Power Calculation Formulas for Highly Myopic Eyes: A Systematic Review and Network Meta-Analysis
Source: J Clin Med. 2026 Jul 24;15(15):5792. doi: 10.3390/jcm15155792 (PMC13467532; doi:10.3390/jcm15155792)
Supplement: Supplementary file 1 [file jcm-15-05792-s001.zip › Additional File S8.pdf]

Additional File S8. Node-split analysis of inconsistency for the percentage of eyes with a refractive prediction error ≤ 0.50 D

**Comparison**	**Direct Estimate**	**Direct SE**	**Indirect Estimate**	**Indirect SE**	**Difference Estimate**	**Difference Standard Error**	**P>z**
Barrett Universal II vs EVO	0.2133043	0.1307684	−0.6956035	0.3664007	0.9089078	0.3874218	0.019
Barrett Universal II vs Haigis	−0.1965362	0.131052	0.3183737	0.30169	−0.5149099	0.3294444	0.118
Barrett Universal II vs Hill RBF 2.0	−0.0891316	0.1621266	0.7261834	0.3058093	−0.815315	0.3466429	0.019
Barrett Universal II vs Hill RBF 3.0	0.1798862	0.2394234	−0.4325887	0.3702831	0.6124749	0.4413781	0.165
Barrett Universal II vs Hoffer Q	−0.8468092	0.3876421	−0.5025258	0.2811897	−0.3442834	0.4919405	0.484
Barrett Universal II vs Hoffer QST	0.2217269	0.2238503	−0.9001283	0.3570906	1.121855	0.4202164	0.008
Barrett Universal II vs Holladay 1	−0.5174894	0.1667397	−0.3389882	0.2593441	−0.1785012	0.3122315	0.568
Barrett Universal II vs Holladay $1_{\text{modified W-K}}$	−0.1280668	0.1446387	0.6192294	0.3245472	−0.7472961	0.3555839	0.036
Barrett Universal II vs Holladay $1_{\text{original W-K}}$	0.0252965	0.1749291	0.5728734	0.3146231	−0.5475769	0.3596506	0.128
Barrett Universal II vs Kane	0.2187244	0.1468269	−0.1242228	0.3399215	0.3429473	0.3690245	0.353
Barrett Universal II vs LSF	−0.5569472	0.2489122	−0.4720556	0.3541641	−0.0848917	0.4347455	0.845
Barrett Universal II vs Pearl-DGS	0.0945145	0.2373252	−0.5179604	0.3740485	0.6124749	0.4413781	0.165
Barrett Universal II vs SRK/T	−0.3369335	0.1384199	−0.0150852	0.2919488	−0.3218483	0.3245163	0.321
Barrett Universal II vs $\text{SRK/T}_{\text{modified W-K}}$	−0.2245968	0.1432583	0.5226993	0.323672	−0.7472961	0.3555839	0.036

Barrett Universal II vs SRK/T$_{original\ W\text{-}K}$	−0.1917141	0.172918	0.3558629	0.3134384	−0.547577	0.3596505	0.128
EVO vs Haigis	−0.0534965	0.1657273	−0.5461395	0.2242883	0.492643	0.2785271	0.077
EVO vs Hill RBF 2.0	0.0132234	0.2688988	−0.0348686	0.2134353	0.0480919	0.3433014	0.889
EVO vs Hill RBF 3.0	−0.3355609	0.2459412	0.4435927	0.3964311	−0.7791536	0.4742655	0.100
EVO vs Hoffer QST	−0.1933208	0.2550038	−0.2550278	0.4219705	0.061707	0.5045303	0.903
EVO vs Holladay 1	−0.2975346	0.2232755	−0.8307114	0.2130684	0.5331769	0.310863	0.086
EVO vs Holladay 1$_{modified\ W\text{-}K}$	0.0168472	0.1835791	−0.3654945	0.2477677	0.3823417	0.3102487	0.218
EVO vs Holladay 1$_{original\ W\text{-}K}$	0.1084515	0.209999	−0.0818283	0.279822	0.1902799	0.3514565	0.588
EVO vs Kane	−0.020142	0.1650855	0.3691349	0.3461748	−0.3892769	0.3846411	0.312
EVO vs LSF	−0.5351454	0.3327799	−0.719453	0.2830832	0.1843076	0.442437	0.677
EVO vs Pearl-DGS	−0.4180544	0.2465016	0.3610991	0.3957805	−0.7791535	0.4742655	0.100
EVO vs SRK/T	−0.4576926	0.1850825	−0.2989387	0.2148896	−0.1587539	0.2834661	0.575
EVO vs SRK/T$_{modified\ W\text{-}K}$	−0.1498684	0.1866005	−0.3315961	0.2548938	0.1817276	0.3178615	0.568
EVO vs SRK/T$_{original\ W\text{-}K}$	−0.0275769	0.2018948	−0.4371211	0.2697379	0.4095442	0.3380719	0.226
Haigis vs Hill RBF 2.0	0.2470603	0.1703157	0.0513773	0.359526	0.195683	0.395729	0.621
Haigis vs Hill RBF 3.0	3.01e-11	0.4195466	0.1524234	0.2532214	−0.1524234	0.4900412	0.756
Haigis vs Hoffer Q	−0.5819199	0.25727	−0.3199585	0.4184415	−0.2619614	0.4976814	0.599
Haigis vs Hoffer QST	−0.2019081	0.4208409	0.0960512	0.2501184	−0.2979593	0.4892825	0.543
Haigis vs Holladay 1	−0.4057426	0.167627	−0.2171026	0.2555587	−0.18864	0.3061102	0.538
Haigis vs Holladay 1$_{modified\ W\text{-}K}$	0.239395	0.1666551	−0.222425	0.2640106	0.46182	0.3117089	0.138
Haigis vs Holladay 1$_{original\ W\text{-}K}$	0.2585381	0.2132098	0.2813807	0.2499075	−0.0228425	0.3272976	0.944
Haigis vs Kane	0.363012	0.1699472	0.0668301	0.2733759	0.2961819	0.321394	0.357
Haigis vs LSF	−0.3502773	0.382736	−0.4385751	0.2457749	0.0882978	0.4536586	0.846
Haigis vs Pearl-DGS	0.4169842	0.4341593	−0.0851023	0.2464199	0.5020866	0.4948016	0.310

Haigis vs SRK/T	−0.1359756	0.1394312	−0.287025	0.3120628	0.1510493	0.3419766	0.659
Haigis vs SRK/T$_{modified\ W\text{-}K}$	0.1119885	0.1684558	−0.2318764	0.2677771	0.3438649	0.3165858	0.277
Haigis vs SRK/T$_{original\ W\text{-}K}$	−0.0796664	0.2064586	0.2424548	0.2481286	−0.3221211	0.3230803	0.319
Hill RBF 2.0 vs Hoffer Q	−0.8585352	0.3881047	−0.6302971	0.3100464	−0.2282381	0.5070597	0.653
Hill RBF 2.0 vs Holladay 1	−0.9010006	0.2476227	−0.3089013	0.211357	−0.5920993	0.3240167	0.068
Hill RBF 2.0 vs Holladay 1$_{modified\ W\text{-}K}$	−0.0374652	0.1848342	−0.3322103	0.3456655	0.2947451	0.3930057	0.453
Hill RBF 2.0 vs Holladay 1$_{original\ W\text{-}K}$	0.136412	0.2128903	−0.1315436	0.3279504	0.2679557	0.3911517	0.493
Hill RBF 2.0 vs Kane	0.2293457	0.2186683	−0.1529753	0.2592815	0.382321	0.3394669	0.260
Hill RBF 2.0 vs LSF	−1.014473	0.3801364	−0.4282017	0.2654713	−0.586271	0.4637948	0.206
Hill RBF 2.0 vs SRK/T	−0.4207745	0.2165098	−0.3213315	0.2269	−0.099443	0.3119414	0.750
Hill RBF 2.0 vs SRK/T$_{modified\ W\text{-}K}$	−0.1529895	0.1849869	−0.3508905	0.3458677	0.197901	0.3916102	0.613
Hill RBF 2.0 vs SRK/T$_{original\ W\text{-}K}$	−0.2090463	0.2123581	−0.0385858	0.3327863	−0.1704605	0.394761	0.666
Hill RBF 3.0 vs Hoffer QST	−0.2063982	0.3024265	0.1606959	0.4539219	−0.3670942	0.5455952	0.501
Hill RBF 3.0 vs Holladay 1	−0.0659237	0.4144212	−0.6226982	0.263804	0.5567745	0.4907927	0.257
Hill RBF 3.0 vs Holladay 1$_{modified\ W\text{-}K}$	0.4411745	0.4337462	−0.1730445	0.2642214	0.6142189	0.511943	0.230
Hill RBF 3.0 vs Kane	0.1574409	0.2525358	0.2051617	0.4338906	−0.0477208	0.5054343	0.925
Hill RBF 3.0 vs Pearl-DGS	N/A	N/A	N/A	N/A	N/A	N/A	N/A
Hill RBF 3.0 vs SRK/T	−0.3285705	0.4125442	−0.2517078	0.2564395	−0.0768627	0.4834733	0.874
Hill RBF 3.0 vs SRK/T$_{modified\ W\text{-}K}$	0.2124253	0.4248489	−0.2184987	0.2642643	0.4309241	0.5020952	0.391
Hoffer Q vs Holladay 1	−0.081591	0.2441749	0.9075141	0.4304791	−0.9891051	0.4940032	0.045
Hoffer Q vs SRK/T	0.4302852	0.256604	0.1320356	0.4183015	0.2982496	0.4967977	0.548

Hoffer QST vs Holladay 1	0.203584	0.4165154	−0.5881738	0.2587569	0.7917577	0.4900981	0.106
Hoffer QST vs Kane	0.2589942	0.3059424	0.2663517	0.3131279	−0.0073575	0.4400631	0.987
Hoffer QST vs Pearl-DGS	−0.1091111	0.2952931	0.3353734	0.4695615	−0.4444846	0.5547127	0.423
Hoffer QST vs SRK/T	0.0813585	0.4207544	−0.2729025	0.2510726	0.3542611	0.4898153	0.470
Holladay 1 vs Holladay $1_{\text{modified W-K}}$	0.297587	0.1952288	0.7025568	0.2413347	−0.4049698	0.3110527	0.193
Holladay 1 vs Holladay $1_{\text{original W-K}}$	0.5709005	0.2322663	0.6729541	0.257086	−0.1020536	0.3453571	0.768
Holladay 1 vs Kane	0.4101624	0.42991	0.6660553	0.1743635	−0.2558929	0.464064	0.581
Holladay 1 vs LSF	0.2113388	0.3133716	−0.2919625	0.2873055	0.5033013	0.4239319	0.235
Holladay 1 vs Pearl-DGS	0.4588446	0.4344671	0.3581618	0.2628215	0.1006828	0.5035124	0.842
Holladay 1 vs SRK/T	0.1697929	0.147185	0.3334706	0.4264722	−0.1636778	0.451782	0.717
Holladay 1 vs $\text{SRK/T}_{\text{modified W-K}}$	0.2001367	0.1962896	0.6120909	0.2412302	−0.4119542	0.3111432	0.186
Holladay 1 vs $\text{SRK/T}_{\text{original W-K}}$	0.4095674	0.2314913	0.3900558	0.2547711	0.0195116	0.3435294	0.955
Holladay $1_{\text{modified W-K}}$ vs Holladay $1_{\text{original W-K}}$	0.1837097	0.1788548	−0.013125	0.4878481	0.1968346	0.5186639	0.704
Holladay $1_{\text{modified W-K}}$ vs Kane	0.0174783	0.1957151	0.4257752	0.2503327	−0.4082969	0.3177886	0.199
Holladay $1_{\text{modified W-K}}$ vs LSF	−0.3663905	0.2413375	−0.9280642	0.3942235	0.5616737	0.4622343	0.224
Holladay $1_{\text{modified W-K}}$ vs Pearl-DGS	−1.23e-12	0.4536487	−0.0956764	0.2627258	0.0956764	0.5242347	0.855
Holladay $1_{\text{modified W-K}}$ vs SRK/T	−0.2179162	0.1775538	−0.3700934	0.2502073	0.1521772	0.3067828	0.620
Holladay $1_{\text{modified W-K}}$ vs $\text{SRK/T}_{\text{modified W-K}}$	−0.0881669	0.1622539	0.1514791	1115.367	−0.239646	1115.367	1.000
Holladay $1_{\text{modified W-K}}$	−0.0322354	0.1773189	−0.2290697	0.4870573	0.1968344	0.5186641	0.704

vs SRK/T$_{\text{original W-K}}$							
Holladay 1$_{\text{original W-K}}$ vs Kane	−0.0875953	0.274711	0.0829814	0.2295733	−0.1705767	0.3578714	0.634
Holladay 1$_{\text{original W-K}}$ vs LSF	−0.2901416	0.3154085	−1.037659	0.3031927	0.7475178	0.4396153	0.089
Holladay 1$_{\text{original W-K}}$ vs SRK/T	−0.464295	0.2339927	−0.3954375	0.230389	−0.0688574	0.3266646	0.833
Holladay 1$_{\text{original W-K}}$ vs SRK/T$_{\text{modified W-K}}$	−0.336704	0.1737899	0.3933843	0.4790779	−0.7300883	0.5076155	0.150
Holladay 1$_{\text{original W-K}}$ vs SRK/T$_{\text{original W-K}}$	N/A	N/A	N/A	N/A	N/A	N/A	N/A
Kane vs LSF	−1.210145	0.382891	−0.4611204	0.2537381	−0.7490242	0.460459	0.104
Kane vs Pearl-DGS	−0.2324617	0.2526575	−0.2801825	0.4345384	0.0477208	0.5054343	0.925
Kane vs SRK/T	−0.4938646	0.216005	−0.3967657	0.2029418	−0.0970989	0.2956203	0.743
Kane vs SRK/T$_{\text{modified W-K}}$	−0.1315474	0.196593	−0.4893421	0.2531407	0.3577947	0.3204809	0.264
Kane vs SRK/T$_{\text{original W-K}}$	−0.2056014	0.2726316	−0.2451548	0.2289052	0.0395534	0.3562375	0.912
LSF vs SRK/T	0.3022226	0.2458176	0.1354959	0.3709735	0.1667267	0.4434019	0.707
LSF vs SRK/T$_{\text{modified W-K}}$	0.5114831	0.24888	0.2120820	0.4007952	0.2994011	0.4736393	0.527
LSF vs SRK/T$_{\text{original W-K}}$	0.3735378	0.3282617	0.5467478	0.3094056	-0.17321	0.4538544	0.703
Pearl-DGS vs SRK/T	-0.7360098	0.4243723	-0.0201164	0.2468149	-0.7158934	0.4846439	0.140
Pearl-DGS vs SRK/T$_{\text{modified W-K}}$	-0.2110225	0.4432905	0.0444513	0.2611832	-0.2554738	0.5122876	0.618
SRK/T vs SRK/T$_{\text{modified W-K}}$	0.2037668	0.1791812	0.1229396	0.2497413	0.0808272	0.3073527	0.793
SRK/T vs SRK/T$_{\text{original W-K}}$	0.3023637	0.2318408	0.1285903	0.2268151	0.1737734	0.3232313	0.591
SRK/T$_{\text{modified W-K}}$ vs SRK/T$_{\text{original W-K}}$	0.1201331	0.1721246	-0.6099552	0.4783834	0.7300883	0.5076156	0.150

SE: standard error; N/A: not applicable; P>z: p value for inconsistency.
