# Supplementary material for: Evaluation of Intraocular Lens Power Calculation Formulas for Highly Myopic Eyes: A Systematic Review and Network Meta-Analysis
Source: J Clin Med. 2026 Jul 24;15(15):5792. doi: 10.3390/jcm15155792 (PMC13467532; doi:10.3390/jcm15155792)
Supplement: Supplementary file 1 [file jcm-15-05792-s001.zip › Additional File S9.pdf]

Additional File S9. Node-split analysis of inconsistency for the percentage of eyes with a refractive prediction error ≤ 1.00 D

Comparison	Direct Estimate	Direct SE	Indirect Estimate	Indirect SE	Difference Estimate	Difference Standard Error	P>z
Barrett Universal II vs EVO	0.0654541	0.2743137	-1.174688	0.7884266	1.240142	0.8257048	0.133
Barrett Universal II vs Haigis	-0.5559118	0.2605363	0.1018669	0.5079004	-0.6577787	0.5738317	0.252
Barrett Universal II vs Hill RBF 2.0	-0.2692157	0.3290913	0.9375610	0.6120426	-1.206777	0.7079817	0.088
Barrett Universal II vs Hill RBF 3.0	-0.1136864	0.5840429	-0.0586010	0.8518264	-0.0550854	1.065104	0.959
Barrett Universal II vs Hoffer Q	-1.050729	0.8056436	-1.163261	0.3941710	0.1125311	0.9239138	0.903
Barrett Universal II vs Hoffer QST	-0.2244406	0.4128151	-1.140786	0.6985946	0.9163456	0.8211224	0.264
Barrett Universal II vs Holladay 1	-0.5718627	0.2832073	-1.525801	0.4132531	0.9539379	0.5062125	0.060
Barrett Universal II vs Holladay $1_{\text{modified W-K}}$	-0.3338867	0.2890041	0.1997201	0.6882990	-0.5336068	0.7586238	0.482
Barrett Universal II vs Holladay $1_{\text{original W-K}}$	0.2763073	0.3615922	0.0201962	0.6636762	0.2561111	0.7337583	0.727
Barrett Universal II vs Kane	-0.0763209	0.3193694	0.8601467	0.6262282	-0.9364675	0.7096738	0.187
Barrett Universal II vs LSF	-0.7554630	0.4113550	-0.9420277	0.6371190	0.1865646	0.8099267	0.818
Barrett Universal II vs Pearl-DGS	-0.0497623	0.6012544	0.0053231	0.8451144	-0.0550854	1.065104	0.959
Barrett Universal II vs SRK/T	-0.8198282	0.2568983	-0.3191095	0.4963924	-0.5007187	0.5630819	0.374
Barrett Universal II vs $\text{SRK/T}_{\text{modified W-K}}$	-0.4627298	0.2810994	0.0708769	0.6855288	-0.5336067	0.7586240	0.482

Barrett Universal II vs SRK/T$_{original\ W\text{-}K}$	-0.3554950	0.3213443	-0.6116062	0.6422162	0.2561112	0.7337582	0.727
EVO vs Haigis	-0.0555124	0.3234167	-0.8526252	0.4154913	0.7971128	0.5212314	0.126
EVO vs Hill RBF 2.0	0.3623717	0.4486800	-0.2127570	0.4376267	0.5751287	0.6248482	0.357
EVO vs Hill RBF 3.0	-0.7065075	0.6570155	1.1161770	0.9097193	-1.822684	1.229375	0.138
EVO vs Hoffer QST	-0.6888535	0.4540828	0.2997674	0.7694928	-0.9886209	0.9406653	0.293
EVO vs Holladay 1	-0.8723837	0.3829182	-0.7655289	0.3958579	-0.1068549	0.5473115	0.845
EVO vs Holladay 1$_{modified\ W\text{-}K}$	0.1903801	0.3419691	-0.8893915	0.4755069	1.0797720	0.5854614	0.065
EVO vs Holladay 1$_{original\ W\text{-}K}$	0.4338405	0.3768956	-0.3030069	0.7168618	0.7368474	0.8024094	0.358
EVO vs Kane	0.1891788	0.3713717	0.1262329	0.5903964	0.0629460	0.6992188	0.928
EVO vs LSF	-0.8540379	0.5788618	-0.7073506	0.4559123	-0.1466874	0.7544290	0.846
EVO vs Pearl-DGS	-0.6527731	0.6701893	1.1699110	0.9086356	-1.822684	1.229375	0.138
EVO vs SRK/T	-0.8279038	0.3613509	-0.4849295	0.3672038	-0.3429743	0.5010043	0.494
EVO vs SRK/T$_{modified\ W\text{-}K}$	-0.1807971	0.3412289	-0.6261429	0.4929223	0.4453459	0.5946335	0.454
EVO vs SRK/T$_{original\ W\text{-}K}$	-0.0905204	0.3517817	-1.0111100	0.5614992	0.9205893	0.6624863	0.165
Haigis vs Hill RBF 2.0	0.4874025	0.3079309	0.1622259	0.6366160	0.3251766	0.6908934	0.638
Haigis vs Hill RBF 3.0	0.4169914	0.9689373	0.2959043	0.5266736	0.1210871	1.077695	0.911
Haigis vs Hoffer Q	-0.8461652	0.3774512	-0.3644843	0.6724151	-0.4816809	0.7994830	0.547
Haigis vs Hoffer QST	0.3189650	0.5694283	-0.2828307	0.4554234	0.6017957	0.7198941	0.403
Haigis vs Holladay 1	-0.5562058	0.2629940	-0.1744824	0.4402127	-0.3817234	0.5148836	0.458
Haigis vs Holladay 1$_{modified\ W\text{-}K}$	0.2532983	0.2960625	-0.0612463	0.4851239	0.3145446	0.5645458	0.577
Haigis vs Holladay 1$_{original\ W\text{-}K}$	0.4995752	0.4287852	0.7927843	0.4477332	-0.2932091	0.5955610	0.622
Haigis vs Kane	0.6368124	0.3342937	0.3036196	0.5020924	0.3331928	0.5903131	0.572
Haigis vs LSF	0.2159099	0.4781843	-0.7899635	0.3865891	1.0058730	0.6120947	0.100
Haigis vs Pearl-DGS	0.0000000	0.8886249	0.5457394	0.5693138	-0.5457394	1.055354	0.605

Haigis vs SRK/T	-0.2652276	0.2281095	-0.4659512	0.5409765	0.2007235	0.5894030	0.733
Haigis vs SRK/$T_{modified\ W\text{-}K}$	0.1482364	0.2888166	-0.2491969	0.4713221	0.3974333	0.5524503	0.472
Haigis vs SRK/$T_{original\ W\text{-}K}$	-0.2387418	0.3592557	0.3424650	0.4202163	-0.5812068	0.5581633	0.298
Hill RBF 2.0 vs Hoffer Q	-0.6170570	0.7022281	-1.3700580	0.4451548	0.7530007	0.8364746	0.368
Hill RBF 2.0 vs Holladay 1	-0.5342518	0.4945042	-1.0689930	0.3593636	0.5347415	0.6024929	0.375
Hill RBF 2.0 vs Holladay $1_{modified\ W\text{-}K}$	-0.3246756	0.3442088	-0.0276109	0.6546758	-0.2970646	0.7350195	0.686
Hill RBF 2.0 vs Holladay $1_{original\ W\text{-}K}$	0.4104781	0.4381170	-0.2012063	0.6217337	0.6116844	0.7566377	0.419
Hill RBF 2.0 vs Kane	0.0935222	0.4040181	0.1348058	0.5872318	-0.0412835	0.7129035	0.954
Hill RBF 2.0 vs LSF	-1.2914340	0.6272501	-0.5664568	0.4620280	-0.7249769	0.8014827	0.366
Hill RBF 2.0 vs SRK/T	-1.0776750	0.3735385	-0.3603942	0.3747923	-0.7172804	0.5098969	0.160
Hill RBF 2.0 vs SRK/$T_{modified\ W\text{-}K}$	-0.4327665	0.3346229	-0.2324952	0.6378727	-0.2002713	0.7123212	0.779
Hill RBF 2.0 vs SRK/$T_{original\ W\text{-}K}$	-0.4118864	0.3797916	-0.4517582	0.6015713	0.0398718	0.7125848	0.955
Hill RBF 3.0 vs Hoffer QST	-0.6847150	0.5943929	0.2813687	0.8476102	-0.9660837	1.016039	0.342
Hill RBF 3.0 vs Holladay 1	-0.3466807	0.9680179	-0.9012042	0.5357258	0.5545235	1.082532	0.608
Hill RBF 3.0 vs Holladay $1_{modified\ W\text{-}K}$	-0.4436645	0.9689092	-0.0642029	0.5508224	-0.3794616	1.091891	0.728
Hill RBF 3.0 vs Kane	0.1303084	0.5762943	0.4318636	0.9303522	-0.3015552	1.107519	0.785
Hill RBF 3.0 vs Pearl-DGS	N/A	N/A	N/A	N/A	N/A	N/A	N/A
Hill RBF 3.0 vs SRK/T	-0.7203238	0.9115647	-0.5868612	0.5237814	-0.1334627	1.009359	0.895
Hill RBF 3.0 vs SRK/$T_{modified\ W\text{-}K}$	-0.4233956	0.9689953	-0.2400215	0.5446771	-0.1833741	1.088306	0.866
Hoffer Q vs Holladay 1	0.0275644	0.3288686	1.2361230	0.6666223	-1.208558	0.7434259	0.104
Hoffer Q vs SRK/T	0.6541211	0.3505553	-0.2642580	0.6336131	0.9183792	0.7365445	0.212

Hoffer QST vs Holladay 1	-0.8419743	0.5458996	-0.0821213	0.4743841	-0.7598530	0.7062085	0.282
Hoffer QST vs Kane	0.9332151	0.6194364	0.3449112	0.5145904	0.5883039	0.8215945	0.474
Hoffer QST vs Pearl-DGS	1.0473490	0.6469448	-0.4706064	0.7828097	1.5179550	0.9896740	0.125
Hoffer QST vs SRK/T	-0.3598382	0.5696808	-0.1752629	0.4547612	-0.1845753	0.7178623	0.797
Holladay 1 vs Holladay $1_{modified\ W\text{-}K}$	0.3393333	0.3858191	0.8866505	0.3665619	-0.5473172	0.5316944	0.303
Holladay 1 vs Holladay $1_{original\ W\text{-}K}$	0.6581221	0.4651659	1.4543100	0.4221111	-0.7961879	0.6065037	0.189
Holladay 1 vs Kane	0.3734312	0.9660060	1.0586020	0.3164495	-0.6851708	1.0137580	0.499
Holladay 1 vs LSF	-0.0772677	0.5088208	0.1499933	0.4273396	-0.2272610	0.6659739	0.733
Holladay 1 vs Pearl-DGS	0.0000000	0.8857717	1.2030090	0.5771600	-1.2030090	1.0572160	0.255
Holladay 1 vs SRK/T	0.1569508	0.2272015	0.1793331	0.7885612	-0.0223822	0.8148267	0.978
Holladay 1 vs $SRK/T_{modified\ W\text{-}K}$	0.0092760	0.3500713	1.0184950	0.3545116	-1.0092190	0.4984912	0.043
Holladay 1 vs $SRK/T_{original\ W\text{-}K}$	0.1359456	0.4021218	0.7852202	0.3959621	-0.6492747	0.5629376	0.249
Holladay $1_{modified\ W\text{-}K}$ vs Holladay $1_{original\ W\text{-}K}$	0.1718805	0.3502340	1.9794230	0.7658523	-1.807542	0.8263219	0.029
Holladay $1_{modified\ W\text{-}K}$ vs Kane	0.4960966	0.3565881	0.0274484	0.5778845	0.4686482	0.6747330	0.487
Holladay $1_{modified\ W\text{-}K}$ vs LSF	-0.2595380	0.3579742	-1.648318	0.6991466	1.3887800	0.8013777	0.083
Holladay $1_{modified\ W\text{-}K}$ vs Pearl-DGS	0.0000000	0.8891351	0.3150819	0.5949600	-0.3150819	1.0698310	0.768
Holladay $1_{modified\ W\text{-}K}$ vs SRK/T	-0.4252622	0.3067216	-0.5450874	0.4341131	0.1198251	0.5254640	0.820
Holladay $1_{modified\ W\text{-}K}$ vs $SRK/T_{modified\ W\text{-}K}$	N/A	N/A	N/A	N/A	N/A	N/A	N/A
Holladay $1_{modified\ W\text{-}K}$	-0.4540612	0.3098683	1.3534810	0.7471429	-1.807542	0.8263221	0.029

vs $SRK/T_{original\ W\text{-}K}$							
Holladay $1_{original\ W\text{-}K}$ vs Kane	-0.1628432	0.5076273	-0.0386590	0.5223148	-0.1241842	0.7265874	0.864
Holladay $1_{original\ W\text{-}K}$ vs LSF	-0.7822660	0.5666593	-1.265870	0.5378735	0.4836044	0.7862050	0.538
Holladay $1_{original\ W\text{-}K}$ vs SRK/T	-1.343351	0.4442928	-0.6081523	0.3980749	-0.7351989	0.5603683	0.190
Holladay $1_{original\ W\text{-}K}$ vs $SRK/T_{modified\ W\text{-}K}$	-0.6845831	0.3399254	0.1380652	0.8698426	-0.8226483	0.9001424	0.361
Holladay $1_{original\ W\text{-}K}$ vs $SRK/T_{original\ W\text{-}K}$	N/A	N/A	N/A	N/A	N/A	N/A	N/A
Kane vs LSF	-1.151842	0.5976150	-0.7994820	0.4670929	-0.3523603	0.7743400	0.649
Kane vs Pearl-DGS	-0.0631927	0.5895368	-0.3647479	0.9281505	0.3015552	1.1075190	0.785
Kane vs SRK/T	-0.9107503	0.4433491	-0.7833174	0.3574609	-0.1274329	0.5563539	0.819
Kane vs $SRK/T_{modified\ W\text{-}K}$	-0.3786687	0.3571601	-0.7656052	0.5316978	0.3869365	0.6342768	0.542
Kane vs $SRK/T_{original\ W\text{-}K}$	-0.4830548	0.4701661	-0.5728196	0.4519754	0.0897649	0.6516554	0.890
LSF vs SRK/T	0.3820150	0.3676646	-0.5675878	0.5838834	0.9496028	0.7085703	0.180
LSF vs $SRK/T_{modified\ W\text{-}K}$	0.6043680	0.3894073	-0.0166172	0.6732318	0.6209852	0.8085443	0.442
LSF vs $SRK/T_{original\ W\text{-}K}$	0.4592932	0.5412245	0.3645831	0.4684694	0.0947101	0.7232343	0.896
Pearl-DGS vs SRK/T	-0.3665673	0.8383846	-0.8201418	0.5654068	0.4535745	0.9908565	0.647
Pearl-DGS vs $SRK/T_{modified\ W\text{-}K}$	0.0000000	0.8889818	-0.4987134	0.5883317	0.4987134	1.0660310	0.640
SRK/T vs $SRK/T_{modified\ W\text{-}K}$	0.4242876	0.2987093	0.1669758	0.4087914	0.2573118	0.5035150	0.609
SRK/T vs $SRK/T_{original\ W\text{-}K}$	0.6278062	0.3760891	-0.0025650	0.3628206	0.6303713	0.5157009	0.222
$SRK/T_{modified\ W\text{-}K}$ vs $SRK/T_{original\ W\text{-}K}$	0.0543021	0.2950088	-0.7683462	0.8527542	0.8226483	0.9001423	0.361

SE: standard error; N/A: not applicable; P>z: p value for inconsistency.
